# Chronic Kidney Disease as Oxidative Stress- and Inflammatory-Mediated Cardiovascular Disease

**DOI:** 10.3390/antiox9080752

**Published:** 2020-08-14

**Authors:** Alina Podkowińska, Dorota Formanowicz

**Affiliations:** 1 Dialysis Center Dravis, Dojazd 34, 60-631 Poznan, Poland; alina.podkowinska@gmail.com; 2 Department of Clinical Biochemistry and Laboratory Medicine, Poznan University of Medical Sciences, Rokietnicka 8, 60-806 Poznan, Poland

**Keywords:** oxidative stress, inflammation, chronic kidney disease, cardiovascular disease

## Abstract

Generating reactive oxygen species (ROS) is necessary for both physiology and pathology. An imbalance between endogenous oxidants and antioxidants causes oxidative stress, contributing to vascular dysfunction. The ROS-induced activation of transcription factors and proinflammatory genes increases inflammation. This phenomenon is of crucial importance in patients with chronic kidney disease (CKD), because atherosclerosis is one of the critical factors of their cardiovascular disease (CVD) and increased mortality. The effect of ROS disrupts the excretory function of each section of the nephron. It prevents the maintenance of intra-systemic homeostasis and leads to the accumulation of metabolic products. Renal regulatory mechanisms, such as tubular glomerular feedback, myogenic reflex in the supplying arteriole, and the renin–angiotensin–aldosterone system, are also affected. It makes it impossible for the kidney to compensate for water–electrolyte and acid–base disturbances, which progress further in the mechanism of positive feedback, leading to a further intensification of oxidative stress. As a result, the progression of CKD is observed, with a spectrum of complications such as malnutrition, calcium phosphate abnormalities, atherosclerosis, and anemia. This review aimed to show the role of oxidative stress and inflammation in renal impairment, with a particular emphasis on its influence on the most common disturbances that accompany CKD.

## 1. Introduction

Chronic kidney disease (CKD), with hypertension and diabetes mellitus being the most common causes [1], has been recognized as a risk factor for cardiovascular disease (CVD) independent of other conventional risk factors. CKD and end-stage renal disease (ESRD) carry a 5- to 10-fold higher risk for developing CVD compared to age-matched controls. The Chronic Renal Insufficiency Cohort Study (*n* = 3885) demonstrated that 33% of studied patients (CKD stages 2–4) had concomitant CVD [2]. Strikingly, in patients with mild to moderate CKD, the incidence of cardiovascular mortality is much higher than the incidence of ESRD. The spectrum of CVD in this population extends from arterial vascular disease to left ventricular remodeling with concentric hypertrophy, left ventricular dilation, and heart failure. 

The dynamic inter-relationship between heart and kidney is well known in the clinics. Cardiorenal syndromes (CRS) have been defined as cardiac and renal disorders in which the acute or chronic dysfunction of one organ can cause acute or chronic dysfunction of the other. There are five subcategories of CRS based on the primary damaged organ (heart or kidney) and progression (acute or chronic). Types 1 and 2 describe acute and chronic CVD scenarios leading to acute kidney injury (AKI) or accelerated CKD. Types 2 and 3 involve AKI and CKD, leading primarily to heart failure. However, acute coronary syndromes, stroke, and arrhythmias may be the result of CVD in these forms of CRS. Finally, type 5 of CRS describes simultaneous damage to the heart and kidneys.

Due to the prevalence of CKD in the population worldwide, in recent years, significant research efforts have been directed to identifying the mechanism of accelerated atherosclerosis and other related disturbances in this group of patients. Traditionally recognized risk factors, such as (1) hypertension, (2) hypercholesterolemia, (3) obesity, (4) and hyperhomocysteinemia, alone do not explain the high frequency of CVD in CKD patients. Therefore, it is emphasized that non-traditional risk factors may be essential here, among which, apart from endothelial dysfunction, vascular calcification, volume overload, oxidative stress, and inflammation are indicated [3]. These last two factors have recently gained considerable support as factors relevant in CVD in the setting of CKD. 

Oxidative stress, according to the definition, is the imbalance of redox equilibrium between oxidants (reactive oxygen or nitrogen species, (ROS or RNS, respectively) and free radicals) and antioxidants. Oxidants, due to their molecular instability (e.g., an unpaired electron), promote oxidation reactions with other molecules, such as proteins, lipids, and DNA, to become stabilized. The main ROS include free radicals with an unpaired electron in an oxygen atom, e.g., (1) hydroperoxide (HO_2_^•^), (2) superoxide anion radical (O_2_^•−^), and (3) the reactive hydroxyl radical (HO^•^). There are also redox signaling agents that do not have an unpaired electron but have significant oxidizing properties such as hydrogen peroxide (H_2_O_2_) or singlet oxygen (^1^O_3_). Oxidative stress reactions also involve oxygen compounds with other elements, both free radicals (organic radicals, e.g., the alkoxy radical (RO^•^) and non-radical compounds (peroxynitrite (ONOO^−^), hypochlorous acid (HOCl) [4]. Four distinct pathways of oxidative stress have been identified: (1) classical oxidative stress, (2) chlorinated stress, (3) nitrosative stress, and (4) carbonyl stress; for a review, see [5]. Fortunately, organisms have an antioxidant system that can prevent or slow the injury to the cells caused by free radicals, which includes antioxidant enzymes and non-enzymatic antioxidants, which reduces the adverse effects of ROS. It should be noted that free radicals and reactive species are essential to human wellbeing, having various regulatory roles in the cells. Under physiological conditions, cells defend themselves against ROS damage through antioxidants that remove free intermediate radicals and inhibit oxidation. 

CKD and progressive renal dysfunction are characterized by an amplification of oxidative stress. It has been pointed by several clinical studies, which revealed a gradual augmentation of levels of different oxidative markers including plasma F2-isoprostanes, advanced oxidation protein products (AOPP), malondialdehyde (MDA), and oxidized LDL (ox-LDL). Oxidative stress is the accumulation of many factors; it is linked to the loss of renal function and/or the modality of renal replacement therapy in dialyzed patients. In this particular group of patients, oxidative stress is somewhat a consequence of higher ROS generation, while a disturbed clearance of pro-oxidant substances due to renal insufficiency as well as the inefficient antioxidant defense system play rather a supplementary role [5]. It should be emphasized that it is not always possible to indicate what is the cause and effect.

An example is hypertension. Oxidative stress in the kidney and vascular tissue causes hypertension, and hypertension promotes oxidative stress, meaning the contribution of oxidative stress and hypertension to the vicious circle. Importantly, oxidative stress, along with inflammation, is a critical component of CKD-related pathologies that can adversely affect the human body, leading to many of the disorders discussed in this review. 

At first, ROS has been shown to affect kidney structure. Next, the importance of increased oxidative stress in CKD has been presented. Then, the role of inflammation in promoting oxidative disorders has been shown. In the next section, the reduced activity of antioxidant mechanisms in CKD has been explained. Finally, the relationship between oxidative stress and atherosclerosis, calcium phosphate disorders, and anemia in CKD has been discussed. The article ends with conclusions preceded by data on selected studies on the assessment of cardiovascular risk in groups of patients with CKD. 

## 2. Redox Homeostasis Disturbances Negatively Influence Kidneys Structure

Redox imbalance negatively affects all of the kidney elements, i.e., the renal circulatory system, glomerulus, renal tubules, and interstitial tissue. 

Oxidative stress damages glomerular microcirculation. O_2_^•−^ causes contraction in vascular smooth muscle cells (VSMC), whereas, in endothelial cells, it impairs endothelial nitric oxide synthase (eNOS) [4]. The latter causes the prevalence of local vasoconstrictors (angiotensin II and endothelin 1) and ultimately leads to glomerular ischemia. 

In turn, in the mesangium, excessive ROS production stimulates signalling cascades associated with protein kinase C (PKC), protein kinase B (PKB/Akt), and c-Jun N-terminal (JNK) kinase, which results in phenotypic transformation into fibroblasts [6]. Besides, ROS stimulate the expression of the receptor gene for transforming growth factor-beta (TGF-β) on the surface of mesangial cells and endothelial cells, which, through the cascade of the small mothers against decapentaplegic (SMAD) protein, results in increased collagen and fibronectin synthesis, leading to glomerular fibrosis [7]. Divergent data describe the effect of nitric oxide (NO) and nitrogen derivatives of ROS on mesangial cells. Some researchers found that these compounds are responsible for the overproduction of the extracellular matrix and apoptosis of mesangial cells [8]. On the other hand, some studies show only a minimally toxic effect of ROS nitrogen derivatives due to the well-developed defense system of mesangial cells as opposed to endothelial cells [9]. However, in the case of long-term persistence of redox imbalance, the dominant process is the increased expression of inducible nitric oxide synthase (iNOS) in mesangial cells [6].

Besides, oxidative stress leads to autophagy and an apoptosis of podocytes [8] and negatively affects the tightness of the glomerular filtration barrier [10]. This phenomenon enhances the recruitment of leukocytes because it leads to (1) the release of chemokines (monocyte chemoattractant protein 1 (MCP-1) and colony-stimulating factor 1 (CSF-1)) and (2) increased expression of intercellular adhesive molecule-1 (ICAM-1). It should be emphasized that neutrophils and monocytes/macrophages are the primary source of ROS; therefore, as a result of their increased recruitment, the redox balance shifts toward oxidation [11].

In the renal coils, the most significant amount of ROS is formed at the proximal tubule due to the high oxygen consumption necessary for ATP production (the ATP-dependent 2Na^+^/3K^+^ pump uses ATP). Proximal tubule cells cannot synthesize glutathione, which further promotes the occurrence of oxidative stress [6]. O_2_^•−^ influences the expression of the Na^+^/H^+^(NHE) anti-porting factor, which inhibits sodium ion reabsorption [6]. On the remaining sections of the nephron, O_2_^•−^ acts in opposition, increasing the absorption of the sodium ion from primary urine into the blood [6,12]. At the level of the ascending arm of the loop of Henle, O_2_^•−^ stimulates the ATP-dependent 2Na^+^/3K^+^ pump responsible for maintaining the sodium gradient and increases the activity of the Na^+^/K^+^/2Cl^−^ (NKCC2) co-transporter. In the distal tubule and the collecting coil, the fixation point is the sodium channel sensitive to amiloride (ENaC), which is located in the luminal membrane of epithelial cells, whose action is stimulated by O_2_^•−^. Ultimately, the total balance of ROS activity on the sodium homeostasis tilts toward sodium retention. It is due to the additional effect that ROS and particularly H_2_O_2_ exert on the tubular glomerular feedback that regulates renal excretion of sodium. H_2_O_2_ also enhances the stimulation of juxtaglomerular cells to release renin, leading to the increased activation of the renin–angiotensin–aldosterone system (RAA) [13].

**Figure 1:**
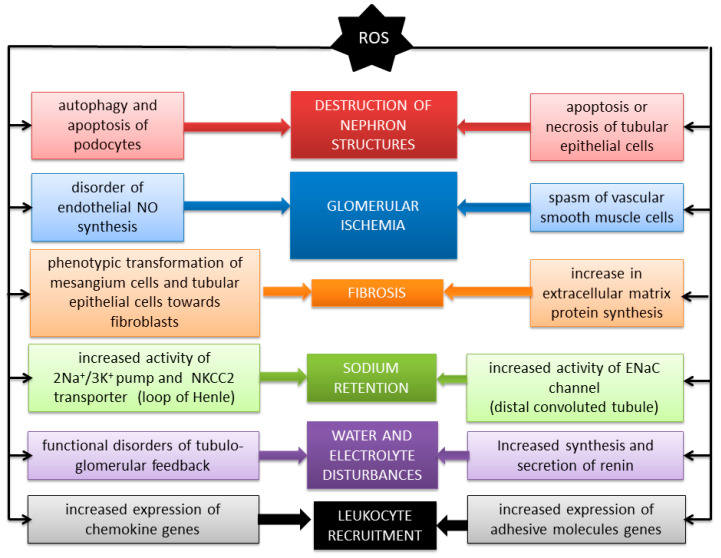
Impact of reactive oxygen species (ROS) on the kidneys.

In the renal tubules, ROS, acting through TGF-β and SMAD protein, similarly to the glomerulus, cause the increased synthesis of extracellular matrix proteins, loss of tubular cell function, and phenotypic transformation toward fibroblasts [14]. Further exposure to oxidative stress leads to cell death by apoptosis (oxidation and activation of caspase 3 and 8) and through necrosis by stimulation of poly [ADP-ribose] polymerase 1 (PARP-1) polymerase [6]. With the loss of function and destruction of the tubules, a deficit of renal excretory capacity, water retention, electrolyte disturbances, and increasing non-breath acidosis are observed. The impact of ROS on the kidneys is summarized in Figure 1.

ROS directly affect individual components of the nephron, leading to the autophagy and apoptosis of podocytes, as well as to apoptosis (by activation of caspase 3 and 8) and necrosis (by activation of the PARP-1 polymerase) of renal tubular epithelial cells. At the same time, under oxidative stress, there is a phenotypic transformation toward fibroblasts in endothelial cells, mesangial cells, and renal tubular epithelial cells. It, together with the overexpression of the TGF-β receptor gene and increased collagen and fibronectin synthesis, leads to fibrosis of the renal parenchyma. Renal blood flow is disturbed as a result of vascular smooth muscle cell contraction and improper functioning of endothelial NO synthase. ROS also affect transport through ion channels in the renal tubules (NKCC2, ENaC), which leads to disturbances in the water and electrolyte balance in the body. Additionally, it is favored by the disruption of regulatory mechanisms such as tubuloglomerular feedback and the intrarenal RAA system. Oxidative stress also stimulates the secretion of chemokines (MCP-1, CSF-1) and the expression of adhesive molecules (ICAM-1), as a result of which an increased recruitment of neutrophils and monocytes/macrophages is observed, which are another source of ROS.

## 3. Excessive ROS Generation in CKD

Oxidative stress in CKD, especially in uremia, may be explained due to increased pro-oxidative activity leading to an excessive generation of ROS. The causes of this are complex and multifactorial, with particular emphasis on the role of increased nicotinamide adenine dinucleotide phosphate (NADPH) oxidase activity (Nox), markedly increased xanthine oxidase, and accompanying mitochondrial dysfunction.

### 3.1. Increased Nox Activity and Role of Angiotensin II (Ang II) in CKD

Griendling et al. for the first time demonstrated that Ang II activates vascular Nox [15]. However, it has been disclosed that the mechanisms by which Ang II regulates Nox are complex and occur at the gene, transcriptional, and post-transcriptional levels and involve numerous intermediate signaling molecules; see [16]. It is of particular interest because Ang II is a complex pleiotropic factor involved in vascular hypertrophy, fibrosis, inflammation, and aging. These effects are mediated by a variety of signaling pathways; of particular interest to these pathways are ROS, which are derived mainly from vascular Nox. By increasing ROS production and activating redox-sensitive transcription factors, Ang II promotes the expression of cell adhesion molecules and induces the synthesis of proinflammatory mediators and growth factors. These molecular and cellular processes facilitate increased vascular permeability, leukocyte recruitment, vascular calcification, and fibrosis, leading to vascular damage and remodeling, as well as premature aging.

Ang II is functionally associated with Nox1, Nox2, and Nox5 and variably with Nox4 in the vasculature. It seems that Nox1 activated by Ang II is essential in VSMC from large arteries, while Nox2 may be more important in low resistance human arteries. The abundance of Nox4 was found to be higher in endothelial cells than in VSMC, at least in some studies. The differences in the regulation of individual Nox isoforms also depend on the type of pathology [16].

Nox1, Nox2, Nox4, and Nox5 expression have been found throughout the kidney, i.e., in glomerular endothelial cells, tubulointerstitial cells, and glomerular cells (mesangial cells and glomerular epithelial cells). Accumulating evidence show that Nox isoforms are a significant source of intrarenal oxidative stress [17] and the stimuli, commonly found in CKD, such as (1) hyperglycemia, (2) hyperlipidemia, (3) hyperhomocysteinemia, (4) hyperuricemia, and (5) obesity, elicit an upregulation of the expression and activity of Nox in metabolic disease-related renal injury [17].

With the increase of our knowledge about Nox functions in various structures and specialized cell types in the kidneys, there is also an increasing awareness that oxidative stress from Nox significantly contributes to many different kidney pathologies. Various studies show the critical roles of Nox-derived ROS in renal fibrosis, especially in conditions of CKD such as diabetic nephropathy [18].

Nox1 elicits the activation of PKC and the phosphorylation of p38 mitogen-activated protein kinase (MAPK) involved in the production of pro-fibrotic mediators, and it takes part in shear-induced outwards vascular remodeling. In vascular pathology, it participates primarily in hypertension and proliferative vascular disease and plays a causal role in the development of diabetic nephropathy [18].

In turn, Nox2, a transmembrane protein bound to a cell membrane or phagolysosome membrane, played a critical role in inflammation, and its activity increases among CKD patients [11,19,20].

Yu et al. reported that Nox5 is the predominant Nox isoform expressed in human renal proximal tubules, and its expression and activity increases in essential hypertension (a small number of individuals slightly limited this conclusion). However, as the study concluded, the increased expression and activity of Nox5 in renal proximal tubules—that is caused, in part, by a defective renal dopaminergic system—may likely be responsible for oxidative stress, which contributes to the pathogenesis of primary hypertension in humans [21]. In turn, Muñoz et al. based on the study of obese Zucker rats (OZR) and their counterparts, i.e., lean Zucker rats (LZR), revealed that the primary role in the oxidative stress of renal vessels and renal endothelial dysfunction in patients with obesity is played by O_2_^•−^ derived from Nox1. In contrast, reduced endothelial Nox4 expression associated with reduced H_2_O_2_ production and H_2_O_2_-mediated vasodilation may hamper the protective function of Nox4 on the kidneys, thus contributing to kidney damage [22].

A unique role is attributed to the Nox4 isoform. It resides partially in the plasma membrane but is localized predominantly to intracellular membranes in mitochondria. H_2_O_2_ derived from Nox4 participates in various physiological functions, including primarily cell proliferation, metabolism, and cell death, but its excessive concentrations induce inflammation, fibrosis, apoptosis, and cell damage. Given the constitutive activity of Nox4, the amount of H_2_O_2_ generated by the enzyme depends on the level of expression. In the physiological states, Nox4 regulates glucose metabolism, affecting Na^+^/glucose transporters (SGLT) and renal gluconeogenesis. It also participates in tubular–glomerular feedback and in the autoregulation of renal blood flow by the myogenic mechanism in the supplying arteriole.

Moreover, Nox4 affects the excretory function of the kidney. It modifies the activity of essential transport structures, such as the (1) Na^+^/H^+^ (NHE) anti-port, (2) 2Na^+^/3K^+^ pump of the basolateral membrane of the tubular epithelium, (3) renal outer medullary potassium channel (ROMK) in the luminal membrane, (4) Na^+^/K^+^/2Cl^−^ (NKCC2) co-transporter, and (5) amiloride-sensitive sodium channel (ENaC) [13]. Whether Nox4 can be harmful or beneficial depends on its abundance, requirements, and the state of the cell at any given time. It has been shown that although glomerular Nox4 expression can be increased in some nephropathies, the loss of tubular Nox4 is a hallmark of all types of CKD. In some studies, the overexpression of Nox4 in the tubular compartment did not induce damage or fibrosis, but instead mildly induced the nuclear factor erythroid 2-related factor 2 (Nrf2) pathway (an emerging regulator of cellular resistance to oxidants). Once activated, Nrf2 can promote a reductive environment by upregulating the expression of several antioxidant enzymes, including enzymes involved in glutathione metabolism. It further proved that Nox4-derived ROS might be not harmful in tubular cells, unlike podocytes [23]. In addition, Nox4 was disclosed to regulate the homocysteine metabolic pathway by promoting cellular protection against oxidant insults. It may explain how low chronic Nox4 activity can facilitate tissue adaptation and heal under stress conditions, and why the complete absence of Nox4 promotes renal fibrosis and oxidative stress found in the unilateral urinary obstruction (UUO) model. Thus, in certain conditions, Nox4 may play a protective role [24].

### 3.2. Increased Xanthine Oxidase (XO) Activity

XO, the enzyme produced in the liver as xanthine dehydrogenase and secreted in the blood, is involved in the uric acid synthesis, and it is a pro-oxidant enzyme promoting the generation of ROS as well. Notably, hyperuricemia has been linked to CVD and mortality in CKD [25] possibly through oxidative stress and endothelial dysfunction. Uric acid has found to be an independent risk factor for renal failure in the earlier stages of CKD. It has a “J” relationship with all-cause mortality in CKD [2]. Ahola et al. assessed uric acid levels at the start of the study and also calculated a genetic risk score for uric acid levels based on 23 single nucleotide polymorphisms (SNPs) that have been shown in other studies to predict uric acid levels. They found that higher uric acid levels were associated with the progression of CKD (defined as worsening to more advanced stages of CKD) in adjusted models with an average follow-up of 7 years [26].

XO produces uric acid as the last step in purine metabolism as well as H_2_O_2_ and ROS. Besides, as a by-product of the purine degradation pathway, XO oxidizes nicotinamide adenine dinucleotide to form O_2_^•−^ and H_2_O_2_. In the endothelium, O_2_^•−^ derived from XO reacts rapidly with NO and forms ONOO^−^, leading to negative feedback on the enzyme. XO is expressed in vascular cells and circulates in plasma, binding to the extracellular matrix of endothelial cells [27]. XO binds to the endothelium, and so far, XO increased levels have only been documented in the arteries of patients on the maintenance of hemodialysis (HD) [28].

Besides, XO activity was found to be an independent predictor of cardiovascular events in CKD and HD patients. Gondouin et al. showed for the first time a correlation between XO and the lipid peroxidation product (MDA) and revealed that XO activity was correlated with antioxidant enzyme superoxide dismutase (SOD) [27]. Scientists show for the first time increased plasma XO activity in HD patients and that XO activity is a strong and independent risk factor for cardiovascular events in CKD/HD patients.

### 3.3. Abnormal Mitochondrial Function in CKD

The kidney receives 20% of the cardiac output and uses 10% of oxygen consumption in the body to perform its primary function by regulating the composition of body fluid by filtering and absorbing substances. The proximal and distal tubular reabsorption, which is mostly driven by adenosine triphosphate (ATP)-dependent active transport, is the most energy-demanding process in the kidney. As a consequence, tubular cells are abundant in mitochondria, and mitochondrial damage and dysfunction carry harmful consequences for many kidney cell functions.

In physiological conditions, mitochondria produce low levels of ROS, which are thought to impact cell signaling processes such as the detection of hypoxia. However, through various pathophysiological stimuli, these organelles become a crucial source of high levels of ROS. Several well-known mechanisms for generating mitochondrial ROS include the consequences of disruption of Fe–S clusters, inhibitory interactions with cytochrome c oxidase, and relative changes in the expression of electron transport chain components [6]. The electron leakage from the respiratory chain means that instead of full oxygen reduction, O_2_^•−^ synthesis occurs. Physiologically, only 1% to 4% of the oxygen undergoes one-electron reduction. At the same time, in conditions of increased oxidative stress, an increased production of ROS is observed at the expense of the correct reduction of molecular oxygen to water. The cause is the deregulation and dysfunction of the mitochondrial respiratory chain. In ESRD, it has shown that in peripheral blood mononuclear cells (PBMC), there is a decrease in cytochrome oxidase activity being the last link in the respiratory chain responsible for electron transfer to molecular oxygen [29]. In turn, many abnormalities in protein genes (COX6C, COX7C, UQCRH, MCAD) related to biogenesis and mitochondrial activity have been observed in renal replacement therapy patients [29,30]. One hypothesis explaining the relationship between the progression of kidney damage and abnormal mitochondrial function assumes that increased ROS production causes a profound inhibition of ATP synthesis, leading to the hypertrophy of the remaining components of the respiratory chain. As a result, further intensification of ROS synthesis is observed, which in the positive feedback mechanism intensifies oxidative stress [30].

An emerging body of evidence indicates that the increased generation of ROS by mitochondria plays a critical role in damaging cellular components and initiating cell death. Importantly, mitochondria regulate constant cell growth, modulating cell aging, leading to irreversible growth inhibition [31]. According to this, oxidative stress and cell aging promote tubular cell apoptosis and mitochondrial dysfunction in vitro, disrupting the regenerative potential of the kidneys.

Apoptotic tubular cells and dysmorphic mitochondria with reduced renal mitochondrial ATP production and an excess generation of O_2_^•−^ were observed in the kidneys of diabetic mice [32]. Besides, studies in two murine models of type-1 and type-2 diabetes showed that glucose-induced mitochondrial ROS generation initiates podocyte apoptosis in vitro and in vivo [33]. Studies based on urine metabolome in patients with diabetic nephropathy confirmed the suppression of their mitochondrial activity [34].

Moreover, experimental studies have shown that mitochondrial damage is crucial in the development and progression of hypertensive nephropathy [35]. In studies of hypertensive rats, a reduced potential of mitochondrial membranes, NOS activity, and cyclooxygenase (COX) activity were observed, suggesting that hypertension coincides with a decrease in mitochondrial function in kidneys [36]. In addition, the proteomic analyses confirmed the existence of differences in the expression of 7 proteins involved in mitochondrial metabolism and oxygen utilization between the hypertensive rats and controls [37].

## 4. Reduction of Antioxidative Activity in CKD

In addition to an increased generation of ROS, the pathogenesis of CKD is associated with insufficient efficiency of antioxidant systems. A profound imbalance between oxidants and antioxidants has been especially suggested in uremic patients on maintenance hemodialysis (HD). However, studies on the activity of antioxidants system, in CKD, especially among ESRD patients on maintenance HD have led to conflicting results.

### 4.1. Superoxide Dismutase (SOD) Is Affected in CKD

Multiple antioxidant systems attempt to protect the kidney from ROS-induced oxidative stress. The first internal enzymes to combat oxidative stress are superoxide dismutase (SOD) isoforms. They catalyze the dismutation of O_2_^•−^ into molecular oxygen and H_2_O_2_. The disturbances in the decomposition of O_2_^•−^ to molecular oxygen enable the generation of subsequent ROS, such as OH^•^, which initiates lipid peroxidation, as well as ONOO^−^ and HClO, which are responsible for the modification of proteins and amino acids [11].

Of the SOD isoforms typically found in the kidney, manganese (Mn)-SOD (SOD-2) is present in the mitochondria, with copper/zinc (Cu/Zn)-SOD (SOD-1 and SOD-3) in the cytosol and the extracellular space, respectively. SOD-1 accounts for up to 80% of the total SOD activity in the kidneys. Interestingly, despite the imbalance in the renal SOD-1 and SOD-2 activity, SOD-2 ablation in mice caused a much more severe pathological phenotype compared to SOD-1 ablation [6]. Other researchers provided evidence that SOD-1 attenuates the renal microvascular response to Ang II by scavenging ROS and maintaining the NO bioavailability. Moreover, it has been shown that SOD-1 is decreased in CKD, showing the lowest protein content in patients treated with HD; however, SOD-1 gene expression was increased in CKD [38]. This increased SOD-1 gene expression may indicate an enhanced protein degradation in patients with CKD and a compensatory increase of SOD-1 gene expression [38].

Several studies have revealed that SOD-1 levels were higher in the serum of HD patients compared to healthy controls [27,39]. Such high levels were associated with a decrease in renal excretion, along with impaired renal function [40]. Besides, Akiyama et al. reported that SOD-1 mRNA levels were also high in the white blood cells of HD patients, which led to increased SOD-1 synthesis [41]. In turn, Futenma et al. examined SOD-1 isomers and showed that despite a decrease in Cu/Zn-SOD tetramers or octamers, a significant increase in SOD-1 levels was observed in patients treated with HD due to an increase in SOD-1 monomers (which are immunologically active but enzymatically inactive) [42]. Thus, it appears that both immune status and enzymatic activity may be necessary for SOD-1 measurements in HD patients, which may also explain the existence of divergent SOD-related data. Besides, Pawlak et al. [40] found in their several studies that serum SOD-1 levels in peritoneal (PD) and HD patients have been associated with several factors, such as (1) age, (2) creatinine clearance, (3) dialysis period, (4) atherosclerosis, (5) vascular damage, (6) hemostasis, (7) coagulation, (8) vascular repair, (9) angiogenesis, (10) inflammation, and (11) hepatitis. They found that an increase in serum SOD-1 levels was associated with decreased renal function with aging and atherosclerosis. In addition, infection, vascular puncture, and hemostasis may be associated with increases in serum SOD-1 levels in PD and HD patients. It should be noted that SOD-1 is primarily used as an antioxidant marker. However, the researchers found that because it is associated with many factors in HD patients, it could serve as a complex marker for atherosclerosis, vascular conditions, and inflammation [43]. It should be emphasized here that the research results regarding SOD are not conclusive. For example, a study by [44] presented no difference in SOD-1 activity between dialyzed and non-dialyzed uremic patients compared to controls.

In opposition to SOD-1 (most of the results showed increased SOD-1 levels in HD), the lower SOD-2 content in mononuclear cells was associated with better survival in HD patients [43]. In uremic rats, Lim et al. [45] discovered reduced SOD-2 in the liver, but Finsch et al. found increased SOD-2 in the kidney [46]. Besides, SOD-2 protein has been shown to be degraded by the ubiquitin–proteasomal pathway [47], whose activity in CKD is enhanced [48]. Krueger et al. found that the SOD-2 protein content showed a J-curve pattern with significantly lower values in the CKD group compared to healthy volunteers (HV) and a progressive reduction up to CKD stage 4, followed by higher SOD-2 protein content in the advanced stages of CKD, i.e., CKD5 with and without HD treatment [49]. The mechanisms underlying this increase have not yet been elucidated. However, it should be noted that higher SOD-2 protein content in ESRD did not influence survival in this group of patients. Given the association with vitamin D metabolism, higher SOD-2 protein content could be associated with more severe uremic disturbances in vitamin D receptor signaling.

Therefore, it is interesting that the increase in SOD-2 protein reported in uremic rats was found to be reversed by vitamin D receptor agonists [46]. Since advanced uremia is a condition of 1,25-dihydroxyvitamin D deficiency, but also a deficiency and dysfunction of the vitamin D receptor, uremic effects associated with the vitamin D receptor may be involved in the SOD-2 changes [50]. These disturbances have been presented in detail in the next section.

Furthermore, SOD-2 polymorphisms were associated with a gradual decline in renal function in patients with type-1 diabetes, suggesting that mitochondrial oxidative stress is a significant path leading to diabetic nephropathy [51]. In addition, SOD-2 expression was reduced in hypertensive rats and was associated with the activation of adrenal inflammation and ROS-generating pathways [52].

In turn, Tan et al. revealed that SOD-3 protects against proteinuric kidney disease in mice [53], and Fujita et al. indicated, on the basis on diabetic mouse models, that the downregulation of renal SOD-1 and SOD-3 may play a key role in the pathogenesis of diabetic nephropathy [54].

### 4.2. Glutathione Peroxidase GPx Changes in CKD

In the case of another enzyme, i.e., glutathione peroxidase (GPx), which is responsible for the detoxification of H_2_O_2_ and organic peroxides, discrepancies regarding individual enzyme isoforms are observed. It should be noted that selenium (Se) is an integral structural component of the active site of red blood cell GPx. Human GPx possesses five isoforms of different organ specificity: GPx1 and GPx4 are found in the renal tubular epithelial cells, GPx3 is poorly detected in the proximal tubules of the kidney, and GPx2 and GPx5 have been not detected in the kidney. GPx1 is the major isoform of GPx expressed in healthy kidney, accounting for 96% of kidney GPx activity [55]. For clarification, in many studies, researchers evaluate GPx1 (E-GPx), which is cellular (also called cytosolic or classical GP) present in red blood cells, and GPx3 (P-GPx), which is extracellular GP present in plasma.

The changes in renal GPx1 expression and activity are considered to play a crucial role in the renal ability to cope with oxidative stress [56]. Since Gpx1 is known to protect against certain types of injury caused by oxidative stress, the induction of oxidative stress of the kidneys during CKD, caused by, e.g., diabetes, should promote the increased expression and activity of this antioxidant enzyme as a protective mechanism. Surprisingly, Haan et al. found in their studies on mice that Gpx1 activity did not increase as a result of diabetic nephropathy. At the same time, there was no compensatory increase in Gpx2-4 levels. However, it was found that along with a decrease in Gpx1 activity, a reduction in CAT levels was observed. It may suggest that Gpx1 may play a role in the induction of CAT gene activity; however, the mechanisms responsible for this are unknown [57]. In contrast to the differences in E-GPx (GPx1) activity in CKD patients, which were found in the reports of various authors, P-GPx (GPx3) activity has been shown to be reduced in CKD consistently by 34–52% as compared with healthy subjects. Several authors have shown a gradual decrease in the GPx3 activity along with the progression of the kidney disease [44,58]. The depression of P-GPx activity in CKD patients indicates that the primary source of the plasma enzyme is the nephron, and its activity might be representative of the residual mass of the kidney. Therefore, it may be suggested that P-GPx activity might be considered as an index of renal function assessment.

Importantly, it should be noted that GPx1 may, on the one hand, protect against oxidative stress. This enzyme was shown to protect red blood cells against hemoglobin oxidation and hemolysis. On the other, an excess in GPx-1 may also have harmful effects due to a lack of essential cell oxidants that cause reductive stress characterized by a lack of oxidants and/or a reduction of excess equivalents. Although this type of stress may seem to be a new concept, it has been known for some time that the lack of cellular oxidants can weaken cell growth reactions; for a better review, see [59]. Decreased selenium (Se) levels and E-GPx activities are common symptoms in CKD patients, especially in the ESRD. Results of studies on the activity of E-GPx in CKD patients are inconsistent.

There is growing evidence from different study types for the existence of the link between GPx-1 polymorphism and CVD outcomes. In the East Asian population, it has been found that GPx1 gene Pro198Leu and Pro197Leu polymorphisms considerably increased the risk of CVD. Large-scale investigations are needed to confirm the results in different ethnicities [60].

In turn, another GPx isoform, GPx3, was found to be affected in the early stages of CKD. Patients with CKD were deficient in this enzyme; however, the clinical consequences of this deficiency were not well understood. Recent findings revealed that mice deficient in GPx3 due to deletion of the GPx3 gene were more susceptible to myocardial micro-clots, impaired ventricular function, and fibrosis in surgically induced CKD. Extreme GPx3 deficiency was also associated with an activation of the myocardial platelet. Thus, GPx3 deficiency was found to be a significant contributor to the development of kidney disease-induced cardiac disease [61]. Besides, it was disclosed that GPx1 increased activity in erythrocytes accompanies decreased plasma GPx3 activity among CKD patients [4,62]. Several studies have reported that there exists an inverse relationship between the degree of vascular disease and the activity of erythrocyte GPx1 [59]. In patients with CVD, the antioxidative enzyme GPx1 seems to protect against adverse oxidative stress effects [63].

Among existing prospective studies, only 1 study has examined the association between E-GPx and myocardial infarction (MI) in a healthy population. A population, prospective, nested case-control study in northern Sweden compared patients with first-ever MI with the control group for Era-Hg (Hg in erythrocytes (Ery-Hg) is often used as an indicator of fish consumption), docosahexaenoic acid (P-PUFA), and E-GPx. Both Ery-Hg and P-PUFA, but not E-GPx, were found to be significantly higher in subjects reporting high fish intake, and it was associated with a low risk of MI. Surprisingly, no association was found between MI and E-GPx, although plasma Se (known to be positively associated with fish intake) in selenocysteine forms the active site of E-GPx [64]. However, the relationship for P-GPx and plasma Se in the Latvian population has been found [65]. In another study, activities of SOD-1, GPx, and catalase (CAT) were not associated with CVD [66].

Disturbances in antioxidant systems that occur from the early stage of CKD and worsen by dialysis provide additional evidence of oxidative stress that may contribute to the development of accelerated atherosclerosis and other long-term complications in uremic patients. HD has been found to influence lipid oxidation more deeply, while PD deteriorates protein oxidation. The activity of antioxidant enzymes is altered by both impaired renal function and dialysis treatment. Interestingly, despite numerous studies in this field, the obtained results are inconclusive, and quite the opposite [67,68,69,70,71,72,73,74,75,76,77,78,79,80,81]. It may be because uremia is a multifactorial process and can be affected by many disturbances.

Selected results of GPx, SOD, and CAT studies in non-dialyzed and dialyzed patients have been presented in Table 1.

### 4.3. Disturbances in the Management of Trace Elements

Another reason for the deficit of antioxidant activity in CKD may be the disturbances in the management of trace elements, such as zinc (Zn) [83,84,85] and selenium (Se) [85,86,87], which are necessary for the proper functioning of antioxidant enzymes. However, on the other hand, some studies do not show significant differences in the concentration of these elements between healthy people and CKD patients [88].

Several mechanisms have been reported to explain the changed status of Se in CKD patients, especially among those on dialysis treatment. These mechanisms include (1) reduced dietary intake, (2) increased loss in urine or dialysis, (3) impaired intestinal absorption, (4) incorrect binding to Se transporters, or (5) drug therapy. It seems very likely that the main reason for the reduced Se concentration in CKD patients is low protein intake and increased protein loss in urine. CKD patients are often advised to limit their protein intake, and it is suggested that reduced protein intake may be the cause of Se deficiency.

In the case of copper ions, both elevated and decreased blood levels were found in patients with ESRD [87,89]. There are also divergent data on the effects of zinc or selenium administration in patients with CKD. The significant impact of trace element supplementation on the increase in antioxidant enzyme activity in this group has not been proven beyond a reasonable doubt. For this reason, the routine administration of zinc or selenium in CKD is currently not recommended [81,90].

Moreover, patients with CKD also revealed a lack of low molecular weight antioxidants. The deficiency concerns more water-soluble compounds such as ascorbic acid and glutathione [91,92] than hydrophobic compounds such as α-tocopherol [91,93]. These deficits, caused by malabsorption due to swelling of the gastrointestinal mucosa and appetite disorders, increase along with the development of hormonal complications found in CKD. It has also been proven that in CKD, the expression of transporters in the cell membrane of enterocytes is reduced, which hinders the absorption of water-soluble vitamins from food [92]. Dietary restrictions necessary for patients with ESRD (restricted potassium diet, low phosphate diet) also increase antioxidant deficiency.

The knowledge of micronutrient disorders in CKD is scarce but has been receiving increased attention recently.

## 5. The Coexistence of Oxidative Stress and Inflammation in CKD

Chronic inflammation is a significant contributor to the promotion of oxidative stress, which is associated with increased oxygen burst response in stimulated monocytes/macrophages and neutrophils. The respiratory burst is an essential element of the body’s defense system, which allows the fight against bacteria, fungi, and parasites. However, under a chronic inflammatory state, there is constant and pathological stimulation of phagocytic cells, which results in an excessive production of ROS. The presence of proinflammatory factors in CKD leads to an increase in oxidative stress, while at the same time, the disturbed redox balance in the body increases inflammation. These two processes drive each other. Thus, the increase in the secretion of the proinflammatory cytokine, such as, e.g., interleukin 6 (IL-6), increases the expression of Nox4, and in turn, the increased expression of Nox4 stimulates IL-6 synthesis [94].

The association between oxidative stress and inflammation is multifaceted. It may exist through different pathways that do not involve classic proinflammatory cytokines, such as tumor necrosis factor-alpha (TNF-α) or IL-6. For example, in the HD group, Uchimura et al. revealed a significant relationship between advanced glycation end products (AGE): pentosidine and N(6)-carboxymethyl lysine (product of both lipid peroxidation and glycation reactions) and the concentration of two proinflammatory cytokines: interleukin 18 (IL-18) and macrophage colony-stimulating factor (M-CSF) [95].

### 5.1. Myeloperoxidase (MPO) Activity in CKD

MPO, a heme-containing enzyme, found in mammalian neutrophils, where it catalyzes the H_2_O_2_, mediates the peroxidation of halide ions and the pseudohalide thiocyanate. Products of these reactions and their secondary metabolites are responsible for killing phagocytized bacteria and viruses. MPO is released from leukocytes into both the phagolysosomal compartment and the extracellular environment upon the activation of phagocytes in peripheral blood, tissues, and fluids [96].

The changes in MPO activity observed in the CKD group are not unequivocal. Emerging evidence suggests that increased MPO activity, via an increased production of diffusing oxidants and NO consumption, maybe an essential risk factor for vascular disease. Several mechanisms by which elevated levels of MPO can promote cardiovascular complications have been described in detail in [97]. Indeed, serum MPO levels have proved to be a strong predictor of cardiovascular events, but in patients without kidney disease [97]. Some researchers showed that MPO activity in the CKD was significantly lower in comparison to controls and decreased as CKD progressed [98]. In this group, the inverse relationship between MPO and urea or creatinine levels was disclosed. Capeillere et al. [38] have suggested that among uremic patients, plasma proteins could be directly oxidized by a mechanism that is MPO-independent, and the generation of AOPP (see the next section) occurs mainly via this MPO independent pathway. They revealed that MPO levels differ significantly between patients before they reach the dialysis stage and those undergoing HD. Moreover, they found that MPO levels were similar in clinically stable CKD patients (but not on dialysis) and healthy controls [23] and even reduced with worsening renal function [98]. However, other studies revealed that patients who were already on renal replacement therapy (HD or continuous ambulatory peritoneal dialysis (CAPD)) had an increase in their basal serum MPO, with the highest values found in the CAPD group. Besides, in HD patients, elevated baseline MPO was associated with inflammation, advanced atherosclerosis, and poor prognosis [99]. Surprisingly, in several studies, basal MPO serum activity in HD patients was in the range of healthy controls [100,101]. Nevertheless, MPO deficiency has been shown to delay CKD progression among 5/6 nephrectomized mice, thus confirming the role of MPO in kidney damage [96,102].

Notably, treatment alone is believed to be partially the cause of the condition. Hemodialysis, as one of the therapeutic procedures performed in patients with renal failure, contributes to the development of chronic inflammation as a result of blood exposure to a biologically incompatible system, activating circulating phagocytes. The hemobio incompatibility depends on the type of dialysis membrane and possible endotoxin contamination of the dialysate. Activated neutrophils and monocytes produce reactive intermediates that contribute to the oxidative imbalance and development of proinflammatory reactions to oxidative stress, in which MPO plays an important role. The increased in MPO and elastase in HD patients was confirmed in comparison with healthy subjects [103]. The production of oxidants begins after the activation of neutrophils, monocytes and macrophages by proinflammatory mediators (IL-1β, IL-6 and TNF-α) when the activity of the NADPH oxidase enzyme (NOX-2) is increased.

### 5.2. Nuclear Factor Kappa-Light-Chain-Enhancer of Activated B Cells (NF-κB Transcription Factor) and Superoxide Dismutase (SOD) in CKD

The relationships between NF-κB, oxidative stress, and inflammation appear to be more complicated than is commonly thought [94,104]. The NF-κB transcription factor, which regulates the expression of proinflammatory cytokine genes, seems to play a vital role in the positive feedback loop between inflammation and ROS production. Under physiological conditions, it is present in the cytosol, in complex with inhibitory proteins (IκB). ROS affect the phosphorylation and degradation of inhibitor of nuclear factor kappa B (IκB), which enables (1) the release of NF-κB, (2) transport to the nucleus, and (3) the transcription of cytokine genes (IL-1α, IL-1β, IL-6, IL-10, TNF-α and β), chemokines (macrophage inflammatory proteins (MIP)), adhesive molecules: vascular cell adhesion molecule 1 (VCAM-1), and intercellular adhesion molecule 1 (ICAM-1), immunoglobulins, and enzymes: cyclooxygenase-2 (COX-2), phospholipase A2, lysozyme.

Moreover, some ROS, such as H_2_O_2_, have been shown to activate NF-κB [104]. Among other things, NF-κB has been found to be responsible for the transcription of the genes of some antioxidant enzymes, e.g., mitochondrial 2 SOD (SOD-2).

### 5.3. Malfunction of the Immune System and Constant Immune Stimulation in CKD Coexisting with Inflammation

The predominance of proinflammatory factors results from the malfunctioning of the immune system and constant immune stimulation, which is superimposed by the reduced renal clearance caused by the increasing loss of active nephrons [105]. Furthermore, the existence of the non-respiratory acidosis, and increased preload in the circulatory system, which is observed with a decrease in renal function, cause an increase in proinflammatory cytokine secretion [106]. In addition, overhydration causes swelling of the intestinal mucosa and an increase in blood–intestinal barrier permeability, which results in the penetration of bacterial toxins from the gastrointestinal tract into the circulation. All these factors appear in patients with diminished renal function, increasing the production of ROS by respiratory burst enzymes [105]. It should be noted that in patients with ESRD, the immune system is particularly affected. In this group of patients, (1) abnormal T helper (Th) lymphocyte maturation, (2) an increase in Th type 1 (Th1)/Th type 2 (Th2) cells ratio, and (3) B cell lymphocytopenia accompanied by (4) normal immunoglobulin levels, (5) disruption of antigen-presenting cells (APC) and an (6) upregulation of scavenging receptors (SR) on phagocytic cells have been disclosed [106].

Since IL-18 stimulates Th1 or Th2 responses depending on the cytokine environment, so its changes may be of crucial importance for the atherosclerotic process.

In our previous study, we focused on the involvement of IL-18 in the immunoinflammatory processes underlying atherosclerosis. IL-18 is one of the essential innate cytokines. It has been disclosed that it can initiate a cascade of proinflammatory cytokines by producing immediate-early inflammatory cytokines such as tumour necrosis factor α (TNF-α) and interleukin 1β (IL-1β). IL-18 was identified initially as an interferon-gamma (IFN-γ) inducer that has been found to support plaque development and instability by contributing to (1) the thinning of the fibrous plaque cap, due to the stimulation of the expression of adhesion molecules on endothelial cells and the major tissue compatibility complex II (MHC II) on macrophages and vascular cells, as well as (2) inhibiting collagen synthesis, which regulates the expression of matrix metalloproteinases upward. Notably, IL-18 not only promotes the synthesis of IFN-γ but also participates in its overall activity. It (3) induces Th1-mediated immune responses during costimulation with other Th1-related cytokines. Alternatively, in synergy with IL-23, it activates (4) Th17 cells to produce IL-17, which is a cytokine that recruits monocytes and neutrophils to the site of inflammation [4]. In the absence of apparent synergies, IL-18 can (5) activate Th2 responses by producing IL-4 and IL-13 [8].

Since the adaptive immune system is involved in the development of atherosclerosis, the deficiency of both T and B cells significantly inhibits the development of atherosclerotic lesions. Most pathogenic T cells in atherosclerosis have a Th1 profile producing high levels of IFN-γ. Therefore, IFN-γ induction may explain the critical role of IL-18 in atherosclerosis. On the other hand, available data suggest that potentially Th2-mediated responses also contribute to the development and progression of atherosclerosis. Therefore, identifying the causes of Th1–Th2 dysregulation would be of great importance for a better understanding of the pathophysiology of atherosclerosis [107].

Although there is no doubt that there exists a close relationship between IL-18 and accelerated atherosclerosis in CKD, only in a few studies was this cytokine evaluated among patients suffering from CKD. In our studies [108], IL-18 was found to be an essential indicator and predictor of cardiovascular death in two-year follow-up among non-diabetic patients suffering from CKD, with a history of acute MI in the previous year [108].

Thus, uremia is associated with a dysfunctional immune state characterized by immunodepression that is likely to contribute to the spread of infections in these patients and immune activation, leading to inflammation that may contribute to CVD. Deterioration of the immune system, whether spontaneous or caused by a predisposition to infection, leads to inflammation and increases the risk of the onset and progression of CVD. It is likely that uremic immune dysfunction significantly contributes to high premature mortality in ESRD patients, mediating cardiovascular and infectious complications, which are two of the most common causes of death in these patients.

### 5.4. Malnutrition–Inflammation–Atherosclerosis (MIA) Syndrome

#### 5.4.1. Malnutrition as a Part of MIA Syndrome in CKD

One of the most severe CKD complications, associated with chronic inflammation, is malnutrition–inflammation–atherosclerosis (MIA) syndrome. Malnutrition in CKD is accompanied by an increased incidence of CVD and rapid progression of atherosclerotic organ damage, according to the term “reverse epidemiology” [62]. Generally, it increases the risk of morbidity, mortality, and overall disease burden in these patients.

##### Hormonal Derangements with Particular Emphasis on Insulin Resistance

There are many causes of malnutrition in patients with CKD: (1) decreased appetite, (2) malabsorption due to overhydration and swelling of the gastrointestinal mucosa, (3) hypercatabolism, (4) loss of protein in the urine, (5) loss of protein during dialysis, and (6) hormonal imbalance, which becomes a characteristic feature; this has been implicated in the suppression of appetite, muscle wasting, and growth impairment in CKD. Chronic inflammation, which is present in CKD, affects the action of many hormones, such as (1) somatostatin (GH), (2) insulin, (3) insulin-like growth factor (IGF-1), (4) ghrelin, and (5) adiponectin. In addition, ESRD patients revealed an impaired balance between proinflammatory leptin and anti-inflammatory adiponectin [109].

Notably, proinflammatory cytokines are responsible for the development of insulin resistance by (1) interfering with insulin (TNF-α, IL-1β), (2) reducing insulin receptor sensitivity (IL-1β), (3) inhibiting glucose metabolism (IL-6), and (4) reducing the number of GLUT4 transporters (IL-6). In addition, there is a disorder of lipid metabolism: an increase in lipolysis with an increased release of free fatty acids (TNF-α, IL-6), abnormal triglyceride metabolism, and stimulation of ectopic lipid deposition (leptin) [110]. Hence, it should be underlined that as CKD progresses, multifactorial insulin resistance develops.

In addition to inflammation, disorders of vitamin D metabolism, and non-respiratory acidosis, one of the essential reasons for insulin resistance is oxidative stress. Under physiological conditions, after insulin binding to the receptor, receptor autophosphorylation and phosphorylation of the insulin receptor substrate 1 (IRS-1) protein occur, which activates the phosphatidylinositol kinase (PI3K) pathway. The final stage consists of the phosphorylation of phosphatidylinositol-dependent protein kinase 1 (PDK1) and phosphorylation and activation of protein kinase B (Akt/PKB). Activated Akt/PKB is responsible, among others, for promoting translocation of the GLUT4 transporter to the cell membrane, which allows glucose to enter the cell. The predominance of proinflammatory cytokines results in a disturbance of signal transduction. The primary mechanism here is, on the one hand, inhibiting the phosphorylation of the IRS-1 protein by TNF-α, and on the other, the synthesis of the suppressor of cytokine signaling 3 (SOC-3) influenced by IL-6 [111]. Increased ROS production results in the inhibition of insulin receptor autophosphorylation and inhibition of IRS-1 protein phosphorylation, which promotes insulin resistance [111]. As a result, in patients with CKD, insulin resistance leads to lipid disorders, endothelial dysfunction, and an increase in blood pressure.

##### Protein–Energy Wasting (PEW)

As it has been proven, along with CKD progression, there is an increased secretion of proinflammatory cytokines, resulting in an increase in resting energy consumption, leading to increased catabolism [112]. It is one of the leading causes of the problems described here.

The International Society of Renal Nutrition and Metabolism (ISRNM) defines protein–energy wasting (PEW) as a “the state of decreased body stores of protein and energy fuels (that is, body protein and fat masses)” [113]. PEW is one of the strongest predictors of mortality in patients with CKD. It is associated with an increase in overall and cardiovascular death risks both in CKD patients not yet on dialysis and in dialysis patients. The prevalence of PEW progressively increases during the decline of kidney function. As a consequence, it has been reported that 40% of patients report symptoms of malnutrition before starting maintenance dialysis treatment. In HD patients, the prevalence of malnutrition varies from 20% to 60% according to which indicators of nutritional status are used [114].

Potential causes of PEW are listed here: (1) increased synthesis of ROS, (2) anorexia, anemia, acidosis, (3) disrupted insulin/IGF signalling, (4) volume overload, (5) oxidative and carbonyl stress, (6) disrupted nutrient intake and prescribed dietary restrictions, (7) nutrient loss during the dialysis session, (8) dialysis treatment-related factors, arteriovenous fistula, dialysis membrane, and (9) comorbid conditions, such as diabetes mellitus, CVD, infections, aging [115].

Other researchers highlighted a unique role of uremic toxins, dialysis techniques, and metabolic dysfunction, showing a vicious circle and a network of inter-relationships between proposed factors.

They emphasized that for PEW, the following are crucial: (1) uremic toxins action that affects both (2) systemic inflammation (IL-6, IL-1β, IL-8, IL-18) and (3) decreased nutrient intake; (4) dialysis techniques stimulating both (5) increased catabolism and systemic inflammation, and (6) metabolic dysfunction: increase in appetite suppression hormones (insulin, leptin, proinflammatory cytokines) causing the decreased nutrient intake and increased catabolism [116].

Particularly noteworthy are the consequences of PEW, such as CVD, vascular calcification, increased mortality, increase in the number of hospitalizations, poor quality of life, decrease in weight and body fat, the decline in albumin, changes in lipid metabolism, sarcopenia, and increased inflammation [115].

##### The Intestinal Microbial Flora Significantly Altered in CKD

The interaction between the intestinal microflora and the kidneys has been confirmed by the term “gut–kidney axis” [117]. High ammonia levels responsible for lowering pH in the gastrointestinal tract, prolonged colon passage, dietary restrictions leading to reduced fiber intake, fluid overload, and medications (such as phosphate binders, proton pump inhibitors, potassium binders, oral iron, and antibiotics) are only a few of the many factors that may underlie the altered composition of the gut microbiota in uremic patients. Disruption of the healthy gut microbiota may lead to intestinal dysbiosis, intestinal barrier dysfunction, and bacterial translocation.

Quantitative studies have shown a reduction in total bacterial count and composition in patients with ESRD. It was discovered that these changes lead to the production and systemic accumulation of proinflammatory uremic toxins, including indoxyl sulfate, p-cresyl sulfate, amines, ammonia, and trimethylamine N-oxide. Moreover, disruption of the intestinal epithelial barrier in CKD patients facilitates the systemic absorption of these toxins. These uremic toxins cause inflammation, endothelial damage, CKD, and PEW. Therapeutic strategies to normalize the gut microbiota in CKD patients can help alleviate chronic inflammation, malnutrition, and other comorbidities [116]. In the studies, patients with elevated systolic blood pressure and CKD revealed altered bacterial composition and decreased bacterial richness [118]. Notably, disruption of normal healthy flora could compromise gut immune function and, in the extreme, create a situation of “immunoparalysis”. The consequence of these effects is systemic cytokine activation, an accumulation of uremic toxins, and endotoxemia [119].

##### Muscles Wasting in CKD

There is a link between CKD progression and metabolic acidosis, inflammation, hormone resistance, and protein catabolism. In the beginning, renal dysfunction reduces proton (H^+^) excretion, causing systemic metabolic acidosis. Acidosis activates the complement systems, RAA, and endothelin-1. These acidosis-mediated symptoms cause CKD to progress, creating a vicious cycle. In turn, acidosis promotes inflammation and tissue resistance to many anabolic hormones, while increasing the activity of catabolic corticosteroids. Protein catabolism generates acidic products, contributing to acidosis in the course of CKD and ESRD. Collectively, these abnormalities lead to a state of protein catabolism, resulting in a persistent negative nitrogen balance, leading to muscle wasting [120].

##### Other Malnutrition-Related Problems in CKD

Hypercytokinemia affects both the gastrointestinal tract, where it slows gastric emptying and the central nervous system, where it inhibits the appetite centre and aggravates depression behaviors. Delayed gastric emptying (gastroparesis) is frequent in patients with CKD, as demonstrated in multiple gastric emptying studies [121]. Elevated levels of gastrointestinal hormones, including gastrin, cholecystokinin, and gastric inhibitory polypeptide, may alter gastric motor function in patients with CKD. Gastroparesis contributes to reducing nutrient intake and is an essential cause of malnutrition in patients with CKD. Dialysis procured and the use of prokinetic agents have been shown to improve the motor function of the gastrointestinal tract in these patients.

CKD predisposes patients to vitamin and mineral deficiencies, which may contribute to comorbidities such as anemia, CVD, and metabolic imbalances. Nevertheless, even with a proper supply of nutrients, a significant number of patients develop malnutrition, which is caused by chronic inflammation.

Although malnutrition is common in dialysis patients in general, the dialysis technique itself may contribute to nutritional deficiencies. For example, hemodialysis patients have higher CRP levels, inflammation, oxidative stress, and increased muscle protein breakdown compared to other CKD patients. It is due to the induction of a cascade of inflammatory pathways when blood comes into contact with the dialyzer membrane. In addition, albumin is lost during peritoneal dialysis, which increases the risk of PEW in these patients. Conversely, less than optimal or insufficient dialysis, can lead to a decreased elimination of uremic toxins, which predisposes patients to develop malnutrition. Crucially, many studies have assessed the effect of a dialysis dose on the nutritional status of dialysis patients and have found an improvement in the nutritional status of patients undergoing dialysis

#### 5.4.2. Inflammation as a Part of MIA Syndrome in CKD

##### Chronic Inflammation

Among the specific characteristics displayed by CKD patients, chronic inflammation plays one of the most critical roles. The inflammatory process is, on the one hand, a body’s protective response to infections and tissue damages, which is characterized by vasodilation and the recruitment of leukocytes, plasma proteins, and fluid to the affected tissue. On the other, if the process is chronic and poorly controlled, such as in CKD, especially in uremia, it may be a maladaptive response that promotes a range of complications. Notably, chronic inflammation is present in a large proportion of the CKD population and is increasingly associated with deterioration in renal function. According to various studies, more than half of patients with CKD stage 3 or higher have elevated levels of CRP [122], with an even higher frequency in patients in ESRD and dialysis patients.

The pathophysiology of chronic inflammation in CKD has not yet been fully elucidated; however, inflammation has been described as a consequence of a multifactorial etiology with interactions with multiple factors that arise in the uremic environment. These include (1) exogenous factors such as dialysis membranes and central venous catheters; (2) cellular factors such as oxidative stress and cell aging; (3) tissue factors such as hypoxia, fluid overload, and sodium overload; (4) microbial factors such as immune dysfunction and intestinal dysbiosis; and finally (5) the retention of uremic toxins such as indoxyl sulfate, advanced glycation end products, and calcioprotein molecules [123]. Among the various comorbidities, particular attention should be paid to the influence of profound changes in the intestinal flora (called dysbiosis), which is typical of CKD.

The systemic inflammation that accompanies CKD has adverse consequences, primarily poor quality of life and increased mortality from CVD and infectious complications. Inflammation is the most critical in uremia. As with other chronic diseases, inflammation in the uremic environment is a consequence of many factors, including comorbidities such as infections. Moreover, in ESRD, inflammation is further exacerbated by uremic immune dysfunction, insufficient renal cytokine clearance, and inflammatory responses to dialysis. It is likely that only by breaking this vicious cycle (s) by acting at several levels of the inflammatory cascade, rather than tackling single causes of inflammation, will it be possible to improve the prognosis of patients with ESRD [114].

Among the numerous inflammatory biomarkers, the interleukin IL-6 seems to be the most reliable predictor of comorbidity and outcomes in CKD [123]. Interestingly, emerging data support the fact that persistent inflammation as part of increased allostatic load plays a significant role in the premature aging phenotype that develops with deteriorating renal function and in the progression of CKD [124].

Among the many inflammatory biomarkers, interleukin IL-6 appears to be the most reliable predictor of comorbidity and outcome in CKD [123]. Emerging data support the notion that persistent inflammation as part of increased allostatic load plays a significant role in the premature aging phenotype that develops with deteriorating renal function [13] and in the progression of CKD [14].

Low-grade chronic systemic inflammation is a condition characterized by persistent, low to moderate levels of circulating inflammatory markers. It has been long associated with coronary heart disease. One should realize that the kidney receives 25% of total blood volume, but without the antioxidant, detoxifying and anti-inflammatory defense mechanisms, it becomes a vulnerable target in the face of persistent “chronic inflammatory aggression”. It is well-known that the increase in the concentration of both acute-phase proteins (C-reactive protein (CRP) and fibrinogen), as well as the inflammatory cytokines (interleukin 1 beta (IL-1β), IL-6 and TNF-α) is proportional to the decrease in GFR and the increase in cystatin C concentration [125].

Systemic or intrarenal inflammation lead to the deregulation of the microvascular response to regulators, supporting the production of tubular toxins. A reduction in nephrons accompanies affected tubules, and CKD begins. Proinflammatory cytokines affect endothelial cells and leukocytes in renal microvessels, which contributes to the local prevalence of proinflammatory factors and ROS. These processes affect the adhesion molecules on the cell surface and disrupt the glycocalyx layer. Endothelial barrier function, activation of the coagulation system, and vasoreactivity are also impaired. These changes may cause irreversible damage to the tubules and damage to the nephron.

Many factors contribute to the setting of the inflammatory status in CKD, including (1) an increased production of proinflammatory cytokines, (2) oxidative stress, (3) acidosis, (4) chronic and recurrent infections, (5) altered metabolism of adipose tissue, and (6) gut microbiota dysbiosis, which is an underestimated source of microinflammation.

The inflammation is strongly emphasized by the fact that the increase of proinflammatory factors (fibrinogen, IL-6) correlates with the mortality of patients in ESRD [126]. Especially in dialyzed patients, a significantly increased level of proinflammatory cytokines (IL-1, IL-6, TNF-α) is maintained, whereas anti-inflammatory interleukins such as interleukins 2, 4, 5 and 12 (IL-2, IL-4, IL-5, and IL-12, respectively) are reduced [105,127]. IL-6 plays a pivotal role, which better than other cytokines reflects the relationship between inflammation and cardiovascular mortality and overall mortality in patients with CKD [96].

##### NLRP3 Inflammasome

Another suggested link between ROS and proinflammatory factors lies in the nucleotide-binding oligomerization domain (NOD)-like receptor family pyrin domain containing 3 (NLRP3) inflammasome. NLRP3 is an intracellular multi-protein complex that recruits pro-caspase-1 via ASC (the adaptor molecule apoptosis-associated speck-like protein containing a C-terminal caspase recruitment domain (CARD)) and then proceeds to cleave the cytokine precursors pro-IL-1β and pro-IL-18 into mature IL-1β and IL-18. Activation of the inflammasome is associated with the leakage of ROS from mitochondria, which may be the result of both CKD and diseases primarily affecting the renal parenchyma (glomerulonephritis or interstitial urethritis) [128]. ROS may also cause the detachment of the thioredoxin-interacting protein (TXNIP) from the thioredoxin (endogenous antioxidant), which allows TXNIP to react with the NLRP3 inflammasome and the activation of the proinflammatory cytokines cascade [94].

##### Kynurenine Pathway

The next link between oxidative stress and inflammation in patients with CKD lies in the kynurenine pathway. In this pathway, tryptophan is metabolized. In the initial stage, tryptophan is converted to N-formylkynurenine by one of two enzymes: tryptophan 2,3-dioxygenase (TDO), which is located in hepatocytes, or indoleamine 2,3-dioxygenase (IDO), which is present in the organs and tissues outside the liver. The catalyst for the reaction carried out by TDO is iron, which oxidizes when combined with an oxygen molecule. However, the proper functioning of IDO requires the presence of reactive oxygen species. Therefore, under oxidative stress, IDO activity increases, and TDO activity decreases.

Proinflammatory cytokines (IL-1, IL-6, IL-8) also stimulate the breakdown of tryptophan with the participation of IDO. Along with the increase in creatinine and urea concentration, increased expression of the IDO gene in monocytes/macrophages and an increase in serum IDO concentration is observed, which results in the accumulation of plasma kynurenine, 3-hydroxykynurenine, kynurenic acid, anthranilic acid, and quinolinic acid [129,130,131]. In addition to the kynurenine pathway, there are also alternative routes of tryptophan metabolism: the serotonin synthesis pathway and the indole pathway, in which compounds classified as uremic toxins are formed (indoxyl sulfate, indole acetic acid, and indoxyl-beta-D-glucuronide).

It should be noted that the products of tryptophan metabolism are classified as aromatic hydrocarbon receptor (AhR) ligands. The aromatic hydrocarbon receptor (AhR) is found in the cytoplasm in association with the chaperone Hsp80. After binding the receptor to the ligand, the complex is shifted to the cell nucleus and dimerized with the aryl hydrocarbon receptor nuclear translocator (ARNT) protein, which allows transcription of a number of genes. It has also been described that—in the positive feedback mechanism—activation of the aromatic hydrocarbon receptor (AhR) by uremic toxins exacerbates chronic inflammation and the production of ROS in endothelial cells, vascular smooth muscle cells, and leukocytes [132].

#### 5.4.3. Atherosclerosis as a Part of MIA Syndrome in CKD

The relationship between oxidative stress and atherosclerosis is complex and multifaceted. Atherosclerosis, underlying CVD, is one of the most severe complications of CKD. The risk of CVD increases along with increasing kidney damage; a decrease in glomerular filtration rate (GFR) by 10 mL/min/1.73 m^2^ increases the risk of CVD by 5%. At the advanced stage of CKD (stage 5), atherosclerotic diseases account for as much as 35–50% of deaths, and in the dialysis group, cardiovascular mortality increases 20 times compared to the general population [133,134]. However, unlike the general population, patients with kidney diseases who are characterized by low body weight, low systolic blood pressure, and low serum TC form a group with an increased incidence of CVD. This phenomenon called “reverse epidemiology” mainly affects dialyzed patients and is caused by the coexistence of two groups of atherosclerosis risk factors.

The first group of traditional risk factors includes (1) age, (2) male sex, (3) overweight or obesity, (4) nicotinism, (5) low physical activity, (6) dyslipidemia (high LDL-cholesterol (LDL-C), low HDL-cholesterol (HDL-C), high triglycerides (TG)), (7) high levels of homocysteine, fibrinogen, and lipoprotein (a); these are accompanied by (8) hypertension and (8) pre-diabetes or diabetes.

Additionally, the second group of factors promoting atherosclerosis appears in CKD, and their dominance over traditional risk factors increases along with the GFR decline. These last risk factors include (1) increase in sympathetic tone, (2) exacerbation of inflammation and oxidative stress, (3) uremic toxin activity, (4) protein–energy malnutrition, (5) calcium phosphate disorders, (6) anemia, (7) endothelial dysfunction, and (8) pro-coagulation. With the gradual decrease in GFR, atypical factors gain an advantage over traditional factors, which causes the existence of the so-called phenomenon of reverse epidemiology of CVD among CKD patients [135,136,137,138]. There seems to be a gradual shift to the uremic lipid profile as kidney function deteriorates, which is further modified by concurrent illnesses such as diabetes and nephrotic syndrome. On the other hand, data from the population-based Atherosclerosis Risk in Communities (ARIC) study support the role of altered lipid metabolism in developing renal dysfunction [139].

The cause of the abnormal lipid profile is the disruption of cholesterol and TG circulation in the body, which leads to atherosclerotic organ damage. The spectrum of dyslipidemia in patients with CKD and dialysis patients is distinct from that of the general population. It involves all lipoprotein classes and shows considerable variations depending on the stage of CKD.

**Figure 2:**
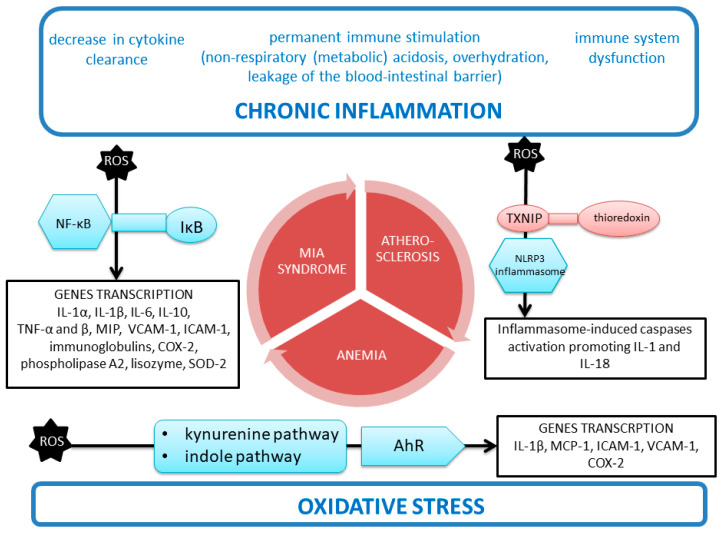
Oxidative stress and chronic inflammatory process in CKD.

The relations between oxidative stress and the chronic inflammatory process in CKD are presented in Figure 2.

Chronic inflammation and oxidative stress—through a positive feedback mechanism—fuel each other, leading to severe complications of CKD, such as MIA syndrome. Hypercytokinemia results not only from a decrease in renal cytokine clearance, but also from persistent immune stimulation caused by non-respiratory acidosis and fluid overload, and from a malfunction of the immune system. One potential link between redox imbalance and inflammation is the NF-κB transcription factor pathway. ROS is responsible for the activation of the NF-κB pathway, which regulates the expression of genes for proinflammatory enzymes and cytokines, chemokines and adhesive molecules. ROS also interacts with the protein complex of the NLRP3 inflammasome, both directly and by affecting the TXNIP protein, which binds to the inflammasome—resulting in the synthesis of proinflammatory cytokines. In patients with chronic kidney disease, redox imbalances promote tryptophan degradation via the kynurenine and indole pathways. Tryptophan metabolites—classified as uremic toxins—are ligands of the aromatic hydrocarbon receptor (AhR), the activation of which is associated with the expression of genes for proinflammatory enzymes and cytokines, chemokines, and adhesive molecules. As a result of the interaction of oxidative stress and chronic inflammation, many complications develop, such as water and electrolyte disturbances, anemia, malnutrition, and atherosclerosis.

## 6. Dyslipidemia in CKD

### 6.1. Lipids, Lipoproteins, and Proteins Modifications

Oxidative stress promotes atherosclerosis by modifying both lipid components, lipoproteins, and proteins. A vicious cycle has been suggested in uremia in which the decreased catabolism of intermediate-density lipoprotein (IDL) and LDL leads to their increased plasma residence time and further modification of the apolipoprotein B (apoB) contained in these lipoproteins by oxidation, carbamylation, and glycation.

#### 6.1.1. Oxidation

The lipid oxidation (peroxidation) process usually is initiated by a highly reactive HO^•^. It can arise as a result of the reaction of HClO (synthesized by MPO) with O_2_^•−^, whose source is the respiratory chain or NADPH oxidase. Further ROS, a peroxide radical (ROO^•^) and an alkyl radical (LO^•^), are formed in the next stages. This process is accompanied by the breakdown of fatty acid residues and the synthesis of aldehydes. It has been shown that the level of MDA (a prominent aldehyde product of lipid peroxidation, as well as of eicosanoid metabolism, that can also form adducts with the lysine residues of apoB) correlates with both GFR and creatinine serum concentration in CKD [140]. MDA has been found to be increased in CKD. Importantly, MDA-modified LDL has also been isolated and characterized from the plasma of patients with CVD.

Although there are several oxidizable components in LDL-C, polyunsaturated fatty acids (mainly arachidonic acid and linoleic acid) of LDL-C lipids are the main targets of oxidizing agents. The first detectable product of lipid oxidation is the phospholipid hydroperoxide. There is also a rearrangement of double bonds to form conjugated dienes. Further oxidation shortens the sn-2 acyl chain to form short-chain aldehyde or carboxyl derivatives. Aldehydes can form adducts with apo B lysine residues, either before or after hydrolysis, from phospholipids by phospholipase A2. 4-hydroxy-2-nonenal (4-HNE) is one of the most abundant aldehydes in oxLDL-C, which derivatizes the thiols and free amino groups of LDL-C apoB and cellular proteins. Importantly, significantly elevated levels of polyunsaturated fatty acid derivatives, such as 4-HNE and 4-hydroxy-2-hexenal (4-HHE), were observed in patients with ESRD [141]. Due to the damaging effects of 4-hydroxy-2-alkenes on vital biological structures (ATP-dependent 2Na^+^/3K^+^ pump, mitochondrial membrane, and respiratory chain), these compounds are now recognized as uremic toxins. Oxidized cholesterol particles (oxysterols) promote the apoptosis of monocytes/macrophages, VSMC, endothelial cells, and hepatocytes [142]. In turn, oxidized phospholipids (ox-PL) increase the expression of adhesive molecules and also contribute to the further intensification of oxidative stress by increasing the activity of NADPH oxidase [143]. In addition to fatty acids, other lipids and also proteins undergo oxidation.

Due to the significantly modified turnover of the lipid subfraction, the residence time of lipoproteins in the circulation of CKD patients is extended. Thus, lipoproteins are at risk for post-ribosomal modification, which includes glycation, oxidation, and carbamylation.

The oxidized LDL-C described in the literature is broadly (and somewhat arbitrarily) divided into two main categories: “minimally modified LDL-C” (MM-LDL-C) and “(fully or extensively) oxidized” LDL-C (ox-LDL-C); for better review, see [144]. The significant difference between these two groups is that the MM-LDL, a proinflammatory and pro-atherogenic lipoprotein, while chemically different from unmodified LDL-C, is still recognized by the LDL-R, and, unlike ox-LDL-C, it is not recognized by SR and thus does not enhance uptake by macrophages.

The extensively modified lipoproteins have reduced affinity for classic LDL-C receptors and are captured by the scavenger receptors (SR) on the surface of macrophages, which are increased in uremia [145]. MPO, which is associated with oxidative lipoprotein modifications, seems to play a unique role here [87]. This enzyme is responsible for the synthesis of hydrochloric acid, cyanic acid, and reactive nitrogen species, which results in lipoprotein modifications. The reaction of hypochlorous acid with apo-B100 tyrosine residues allows LDL-C to bind to the atherogenic lectin-like oxidized low-density lipoprotein receptor 1 (LOX-1) [146]. The cyanic acid tautomer, isocyanic acid, reacts with the LDL-C amino groups, and the resulting carbamylated LDLs are recognized by the SR of phagocytic cells [147]. In turn, nitrated LDL-C derivatives bind to the CD36 scavenger receptor, which leads to the transformation of monocytes/macrophages into the foam cells [148]. In CKD, SR such as SR-A1, SR-A2, SR-BI, and CD-36 play a crucial role in the development of atherosclerosis. The expression of their genes increases as GFR decreases [149]. Modified lipoproteins are not recognized by physiological receptors on the surface of hepatocytes, but they bind to SR on the surface of monocytes/macrophages [150].

The accumulation of ox-LDL-C in phagocytic cells leads to damage to the respiratory chain enzymes and an increase in the leakage of ROS from mitochondria, which further intensifies oxidative stress. Besides, ox-LDL-C uptake by SR is not regulated by cholesterol, resulting in cholesterol/macrophage overloading of monocytes/macrophages. They are transformed into foam cells and form fatty streaks on arterial walls. In the final stage, foam cells undergo apoptosis and cholesterol release, which results in the formation of the necrotic–lipid core of the atherosclerotic plaque [150]. The high affinity for macrophages causes cholesterol accumulation and the formation of foam cells in the walls of the vessels, which ultimately leads to the development of accelerated atherosclerotic plaques.

Pawlak et al., in their multiple regression analysis, found that ox-LDL-C levels and the ox-LDL-C/LDL-C ratio were not raised in uremic patients during dialysis treatment in comparison to controls. Both the ox-LDL and ox-LDL-C/LDL-C ratio were independently correlated with lipid profile but not with other oxidative stress markers. These results suggest that ox-LDL-C levels and the ox-LDL-C/LDL-C ratio can serve as the markers of lipoprotein abnormalities rather than the markers of oxidative stress in the population of dialyzed patients. They confirmed increased ox-LDL-C/HDL-C, total cholesterol (TC), and TC/HDL-C ratios as the parameters independently and significantly predicted ox-LDL-C in dialyzed patients. They suggested that ox-LDL-C levels in peripheral circulation might be associated with a reduced formation of serum HDL-C [39].

Recently, electronegative low-density lipoprotein (LDL-C(−)), a minor modified fraction of LDL, has been found in blood [151]. It comprises 3–5% of total plasma LDL-C (increased in some pathologies), being a heterogeneous population of LDL-C particles modified by various mechanisms sharing as a standard feature increased electronegativity. Modification by oxidation is one of these mechanisms. LDL-C(−) has inflammatory properties similar to those of ox-LDL-C, such as inflammatory cytokine release in leukocytes and endothelial cells. However, in contrast with ox-LDL-C, LDL-C(−) also has some anti-inflammatory effects on cultured cells. The global effect of LDL(−) is a consequence of the combination of its inflammatory/anti-inflammatory properties. Chang et al. discovered that early CKD is associated with an increase in LDL-C(−) that promotes cardiac relaxation dysfunction in vivo. This change disrupts the cardiac isoform of the sarco/endoplasmic reticulum Ca^2+^ ATPase (SERCA2a)-regulated calcium homeostasis, which is a probable mechanism underlying cardiorenal syndrome [152].

Under the influence of ROS, plasma not only lipids but also proteins are oxidized, resulting in the formation of advanced oxidation protein products (AOPPs). Their dominant component is albumin, in which, as a result of oxidation, the formation of dityrosine bonds and disulfide bridges and carbonylation of amino acid residues [153] take place. AOPPs show a high affinity for scavenger receptor class B type 1 (SR-BI) and compete with HDL-C2 for binding to this receptor on hepatocytes. AOPPs also inhibit the expression of the ABCA1 gene, which is responsible for the transmission of cholesterol from cells to HDL-C3. As a result, cholesterol reverse transport is disturbed, and dyslipidemia develops [154]. Serum AOPP are new markers of protein damage induced by oxidative stress. Conti et al. evaluated serum levels of AOPP in a cohort of patients with diabetes and hypertension, with or without renal complications, compared with healthy controls [155]. They found increased levels of AOPP in diabetic patients compared to healthy subjects, although not significantly. However, AOPP was higher and more significant in patients with diabetic nephropathy and CKD secondary to diabetic nephropathy (stages 2–3) than in diabetic patients without nephropathy or control. Patients with hypertension and renal impairment secondary to hypertension also had significantly higher AOPP serum levels than the control group. Moreover, no significant differences were found in mean AOPP concentrations among patients with diabetes and hypertension. The researchers suggested that assessing AOPP in patients with diabetes and hypertension could be significant in predicting the onset of renal failure and opening up a new perspective on the uptake of antioxidant molecules to prevent CKD under these conditions [155].

#### 6.1.2. Glycation

Glycation is a non-enzymatic reaction that proceeds via the formation of a Schiff ’s base, which may then undergo an Amadori rearrangement and form a stable keto-amine link with the exposed lysine residues of a protein, such as apoB in LDL, to produce an early glycation product.

Glycated lipoproteins, including very low-density lipoprotein cholesterol (VLDL-C), LDL-C, and also HDL-C, are believed to contribute to atherosclerotic CVD by impairing lipoprotein metabolism [156] Glycated lipoproteins are catabolized more slowly. For example, glycated LDL-C is not cleared by the physiological pathway of the LDL receptor (LDL-R) and can be taken up by macrophages that form foam cells. The combination of glycation and oxidation that generally coexists in vivo is referred to as glycoxidation. Even though molecular oxygen and non-glycative free radical generating processes are absent in vivo, glycation is accompanied by some degree of oxidation [157].

The glycation of apolipoprotein hinders their removal from the circulation, which contributes to the development of dyslipidemia [158]. Advanced glycation end products (AGEs) are formed as a result in part from the non-enzymatic reaction between reducing sugars and free amino groups of lipids and proteins, which was initially characterized as the browning reaction by the Maillard reaction. It has also been observed that in cultures of endothelial cells, AGEs cause a decrease in the synthesis of vasodilatation factors, disturb the normal maturation and differentiation of endothelial progenitor cells [159], and interfere with the secretion of vasodilatation and vasodilatory factors [160].

ApoB100-containing lipoproteins (as VLDL-C, intermediate-density lipoprotein (IDL-C), LDL-C, and lipoprotein (a) (Lp (a), an LDL-like lipoprotein consisting of apo(a) that is covalently bound to an LDL particle), are potentially atherogenic. Their glycation might explain their atherogenicity.

LDL-C glycation has been particularly studied. It occurs mainly due to the non-enzymatic reaction of glucose and its metabolites with the free amino groups of lysine, in which LDL-C is abundant [161]. Higher levels of glycated LDL-C (g-LDL-C) occur in people with diabetes than in non-diabetic people, but even in the latter, there is generally more circulating glycated LDL-C than oxidatively modified LDL-C. The oxidation and glycation of LDL-C are likely at least partially interdependent, but both prevent uptake via the LDL-R and promote the uptake of the macrophage scavenger receptor. Recognizing that LDL-C glycation is at least as crucial as oxidation in atherosclerosis can lead to a better understanding of the mechanism and how to prevent it [162].

The glycation and oxidation of LDL appear to be intimately associated. Small dense (Sd)-LDL-C, which is known to be most closely associated with atherogenesis and is more susceptible to glycation than more buoyant LDL. sd-LDL and glycated (g)-LDL have been found to be specifically associated with CVD. Recently, a specific pattern of increased ox-LDL-C and sd-LDL-C with worsening CKD has been described [163], and sd-LDL levels have been associated with mortality [164]. It is worth noting that these studies were conducted in typical adult CKD patients over the age of 60, in whom common Framingham risk factors may interfere with the lesions specific to CKD. Hence, Filler et al. evaluated young adult and pediatric patients since they have shorter exposure to Framingham-type risk factors. They determined whether younger CKD patients have the same sd-LDL and g-LDL-C pattern as adults with CKD. They demonstrated that only TG and sd-LDL were associated with CKD stages among young patients without Framingham-type CVD risk factors and suggested that lowering sd-LDL levels may be a potential target to improve the long-term CVD risk in young patients with CKD [165].

### 6.2. Lipids Profile in CKD

Apart from significant qualitative changes, such as oxidization and modification to sd-LDL, which render the particles more atherogenic (see the previous section), quantitative differences in lipoproteins among CKD patients can be observed.

Dyslipidemia, occurring along with the progression of CKD, is characterized by (1) hypertriglyceridemia, (2) decrease in HDL-C and apolipoprotein (apo) AI (apoA-I), (3) increase in IDL-C and chylomicron remnants, followed by (4) reduced apoC-II/apoC-III ratio. ApoC-II is an activator of lipoprotein lipase (LPL), whereas apoC-III is an inhibitor of LPL. The decreased apoC-II/apoC-III ratio is usually due to a disproportionate increase in plasma apoC-III [140,145,166,167].

#### 6.2.1. Triglycerides (TG)

Plasma TG begins to rise in the early stages of CKD and show the highest concentrations in nephrotic syndrome and dialysis patients, especially in PD. The accumulation of TG is a consequence of both the high production rate and the low fractional rate of catabolism. An increased production of TG-rich lipoproteins is probably the consequence of impaired carbohydrate tolerance and increased liver VLDL-C synthesis. The reduced fractional catabolic rate is likely due to the reduced activity of the LPL and the hepatic triglyceride lipase, which have the primary physiological function of cleaving triglycerides into free fatty acids (FFAs) for energy production or storage. Besides, downregulation of the hepatic LRP prevents the proper removal of IDL-C and chylomicron remnants, so they accumulate in the blood [140].

Additional problems in the dialyzed group result from the therapeutic procedures used during dialysis treatment. It was found that frequent heparinization during HD decreases—along with the influence of other plasma lipase inhibitors—the fractional catabolic rate of triglycerides, leading to an increased level of serum TG [146]. Moreover, an increase in the plasma apoC-III/apoC-II ratio is believed to be the cause of the decreased uremic lipase activity. ApoC-II is an LPL activator, while apoC-III is an LPL inhibitor. The increased apoC-III/apoC-II ratio is usually caused by a disproportionate increase in plasma apoC-III. Impaired plasma lipase activity in uremia may also be caused by a decrease in LPL synthesis as a result of secondary hyperparathyroidism (see in the next sections) or decreased insulin levels [168].

#### 6.2.2. High-Density Lipoprotein Cholesterol (HDL-C)

HDL-C plays an essential role in reverse cholesterol transport, which shuttles cholesterol from peripheral cells to the liver: an important step that relieves the peripheral cells from cholesterol burden. HDL precursor particles are secreted as disc-shaped structures by the liver and intestine and can absorb free cholesterol from cell membranes, which is a process that is mediated by ATP binding cassette transporter 1, apoA-I, and apoA-IV. ApoA-I is the primary apolipoprotein of HDL-C and activates the enzyme lecithin: cholesterol acyltransferase (LCAT), which esterifies free cholesterol to allow more efficient packaging of the cholesterol for transport. By the acquisition of additional apolipoproteins, cholesteryl esters, and TG, HDL-C3 particles are transformed into larger HDL-C2 particles.

HDL-C usually protects against plaque formation and progression by mediating reverse cholesterol transport and by exerting potent antioxidant, anti-inflammatory, and antithrombotic actions. However, in the presence of the systemic inflammation found in CKD, HDL-C is transformed into a pro-oxidant and proinflammatory agent. Both qualitative and quantitative changes in HDL-C have been described in patients with CKD. In particular, HDL-C abundance decreases, and HDL-C acquires proinflammatory properties instead of the usual anti-inflammatory role.

Generally, patients with CKD have reduced plasma HDL-C concentrations compared with non-uremic individuals. Furthermore, the distribution of HDL-C sub-fractions is altered. The decreased ability of the HDL particles to carry cholesterol leads to an impairment in the reverse cholesterol transport from peripheral cells to the liver, thereby burdening the vasculature with cholesterol and promoting atherosclerosis.

Oxidative modifications of apoA-I reduce the affinity of HDL-C3 for the ABCA1 transporter responsible for transmitting cholesterol from cells to HDL-C3. In addition, the conversion of HDL-C3—containing free cholesterol—to HDL-C2, which is rich in cholesterol esters, is inhibited due to decreased activity in cholesterol acyltransferase (LCAT). The LCAT activity is affected by the decreased synthesis of this enzyme in the liver and the inactivation by ROS. In the context of ROS, the formation of 3-chlorothyrosine, as a result of increased MPO activity, leads to a decreased LCAT activity in HDL-C3 [140]. Under physiological conditions, the conversion of HDL-C3 to HDL-C2 allows the transmission of cholesterol from peripheral tissues to the liver. In CKD, the formation of HDL-C2 recognized by the scavenger receptor class B type 1 (SR-BI) on the surface of hepatocytes is disturbed, and the process of reverse cholesterol transport is affected. Instead, endocytosis and the degradation of HDL-C3 in hepatocytes is mediated through ATP synthase β chain [166].

Furthermore, infection-associated or uremia-associated inflammation in ESRD might convert HDL from an antioxidant into a pro-oxidant particle [166].

##### Paraoxonase (PON)

Besides the impaired function of HDL-C, as the cholesterol carriers, patients with CKD have reduced activity of the HDL-associated enzymes, such as paraoxonase (PON) and P-GPx [167], which may be responsible for the impaired anti-oxidative and anti-inflammatory function of their HDL-C. PON occurs in three isoforms, two of which (PON-1 and PON-3) are associated with HDL-C. This enzyme is responsible for inhibiting lipid peroxidation, as well as removing H_2_O_2_ and cholesterol from the monocytes–macrophages axis. When plasma PON activity is reduced, it predisposes the HDL-C and also low-density lipoprotein cholesterol (LDL-C) particles to oxidation. The decreased PON-1 activity in patients with CKD may result from the direct inactivating effect of uremic toxins and advanced glycation products (AGEs) [169]. Notably, low levels of serum PON-1 have been associated with obesity-related disorders and CVD [170]. Several mechanisms have been proposed to explain PON-1 changes in CKD; mostly, they relied on (1) the role of uremic toxins (AGE, acrolein (a major component of cigarette smoke, having several endogenous sources)) and (2) HDL-C composition and PON-1 association [171]. Notably, results from multiple case-control studies presented that renal failure was associated with deficient PON-1 activity independently of HDL-C changes [171]. Interestingly, an association between PON-1 activity recovery after dialysis has been found with creatinine changes, AGE, and acrolein, suggesting that uremic toxins may play a mechanistic role in PON-1 inactivation.

##### Ischemia-Modified Albumin (IMA)

It has been discovered that PON-1 and ischemia-modified albumin (IMA)—a biomarker of cardiac ischemia—could predict CVD-related mortality in ESRD, and it may be useful for preventive strategies for CVD. Kotani et al., in their review, proposed a possible interaction between PON-1 and IMA in ESRD [172].

Damage to a specific motif (DAKK motif) at the N-terminus of albumin reduces the binding capacity of transition metals. The effect is related to the circulation of albumin in the ischemic vessels of the capillaries, which occurs in CVD, especially in acute coronary syndromes. Researchers have suggested that the formation of IMA can occur not only due to acute but also chronic oxidative stress. Recent studies have shown significantly higher levels of IMA in patients with ESRD compared to controls. The association between PON-1 and IMA in a small cohort of HD patients revealed that PON-1 levels were significantly and inversely correlated with IMA concentrations, while no clear correlation was found in controls. This inverse correlation can be explained in part by the suggestion that the low PON-1 activity in these patients causes increased oxidative stress, leading to the formation of IMA. Therefore, the simultaneous monitoring of PON-1 and IMA in serum may be another useful tool as a prognostic biomarker for patients with CVD in ESRD [172].

#### 6.2.3. Very Low-Density Lipoprotein Cholesterol (VLDL-C)

The disrupted HDL-C metabolism leads to the impaired distribution of apo-C and apo-E, which prevents the proper maturation of VLDL-C and chylomicrons. ApoE is a ligand that is critical for the removal of the particles from the circulation by binding to multifunctional lipoprotein receptor-related protein (LRP) and perhaps other receptors on the vascular wall. Therefore, the arterial wall is exposed to high plasma levels of remnant lipoproteins for a prolonged time, which may predispose to atherogenesis. Moreover, the decrease in LPL activity and downregulation of the VLDL-C receptor in muscles and fat tissues inhibits the hydrolysis of the triglycerides, with the subsequent release of free fatty acids from VLDL-C and chylomicrons [145,166].

In turn, an additional decrease in liver lipase activity makes it difficult to enrich IDL in cholesterol esters and remove triglycerides [167].

#### 6.2.4. Low-Density Lipoprotein Cholesterol (LDL-C) and Lipoprotein a (Lp (a))

LDL-C metabolism is also affected in CKD, and this is mainly in the course of oxidative stress. Ox-LDL has been widely presented in the previous section. Although LDL is not usually elevated in patients with CKD, LDL particles tend to be smaller, denser, and more atherogenic in their form.

Many studies have revealed increased levels of small dense LDL-C (sdLDL-C) in non-dialysis-dependent CKD patients in comparison with healthy subjects, indicating sdLDL-C as an essential emerging risk factor for CVD. sdLDL-C particles have a small size, which enables them to penetrate easily into the arterial wall. Moreover, due to their high affinity for proteoglycans in the arterial wall, leading to prolonged residency in the sub-endothelial space, they are strongly atherogenic. Moreover, it should be noted here that sdLDL-C, which is known to be most closely associated with atherogenesis, is more susceptible to glycation than more buoyant LDL-C (see the previous section).

On the other hand, in the study conducted by Savic at al., the concentration of sdLDL-C was the highest in the pre-dialysis group and the lowest in HD patients. Furthermore, these researchers disclosed an association of pro-atherogenic sdLDL-C subclasses with markers of inflammation, such as galectin-3. CKD patients with increased galectin-3 concentrations had significantly higher relative proportions of cholesterol in sdLDL-C (% sdLDL-C) than their counterparts with lower galectin-3 levels.

In this mysterious interaction between dyslipidemia and inflammation, galectin-3—a molecule with diverse biological activity—can play an important role. It has been shown to act proinflammatory by activating cell chemotaxis and adhesion [173]. Galectin-3 has also been shown to be a receptor for AGEs, as well as for modified LDL particles, which further confirms its suggested role in promoting atherosclerosis. On the other hand, galectin-3 can perform an atherosclerotic function by increasing the removal of modified lipoproteins. The relative proportion of sd-LDL-C was shown to be an independent determinant of galectin-3 concentration. Probably, proportions of sd-LDL-C are related to the stages of CKD [173]. It is essential because sd-LDL-C was disclosed to be highly susceptible to oxidation.

Despite lower mean LDL-C levels, HD patients have significantly decreased the cholesterol/triglyceride ratio, reflecting the domination of sd-LDL-C. Other studies have also shown that HD patients have elevated Lp (a) and ox-LDL forms, even when their LDL levels are normal [174].

Lp (a) is considered to compete with plasminogen for binding to plasminogen receptors, fibrinogen, and fibrin. Thus, by the inhibition of fibrinolysis, it promotes thrombogenesis and is highly associated with the occurrence of CVD in the general population. It should be noted that serum Lp (a) levels depend on the size of the apo (a) isoform and are strongly genetically determined by the apo (a) gene. Patients with large apo (a) isoforms, rather than small ones, usually have elevated Lp (a) levels at an early stage of CKD, even before GFR is significantly reduced. Various studies, both in healthy individuals and patients with CKD, have shown a strong and adverse relationship between the size of the apo (a) isoform and the serum Lp (a) level. Serum Lp (a) levels depend on the size of the apo (a) isoform and are strongly genetically determined by the apo (a) gene. It means that people with mostly low molecular weight apo (a) isoforms have, on average, higher plasma Lp (a) concentrations [174]. In vivo studies, using stable-isotope techniques, elucidated the mechanism of increased Lp (a) plasma levels in HD patients. The production rates of apo (a) and apoB, the two apolipoproteins contained in Lp (a), were normal compared to controls. However, in HD, the fractional catabolic rate of apo (a) and apoB was significantly reduced compared to the controls. It resulted in a significantly longer plasma residence time of almost nine days for apo (a) compared with only 4.4 days in controls. This reduced clearance is probably due to a loss of renal function in HD patients [175].

It should be noted that malnutrition and inflammation are also associated with high plasma levels of Lp (a) in HD. However, an increase in plasma Lp (a) can be observed even in patients with normal plasma C-reactive protein. Prospective studies have shown that the small apo (a) isoform is an even more reliable predictor of total and cardiovascular mortality in patients with CKD than total plasma Lp (a).

#### 6.2.5. Total Cholesterol (TC)

The relationship between total plasma cholesterol and mortality in HD patients was found to be U-shaped. The group with TC levels between 200 and 250 mg/dL was found to have the lowest risk of death, whereas those patients with levels > 350 mg/dL had a relative risk of 1.3-fold and those with levels < 100 mg/dl had a relative risk of 4.2-fold. Interestingly, another study on a group of 1167 HD patients found that among those with low plasma albumin levels (up to 3.9 g/dl) [145], low plasma TC levels were also associated with increased all-cause mortality. This surprising relationship was confirmed in the CHOICE Project, which showed a non-significant negative association of cardiovascular mortality with plasma TC as well as non–HDL-C levels in the presence of inflammation and/or malnutrition [176]. These observations are consistent with the hypothesis that the inverse relationship of TC levels with mortality in dialysis patients is due to the cholesterol-lowering effect of malnutrition or systemic inflammation, or both of them, but neither is related to the protective effect of high TC levels.

#### 6.2.6. ApoA-IV

ApoA-IV is involved in a broad spectrum of physiological processes such as lipid absorption and metabolism, glucose homeostasis, protection against atherosclerosis, platelet aggregation and thrombosis, and food intake. ApoA-IV deficiency is associated with atherosclerosis and diabetes, which renders it as a potential therapeutic target for the treatment of these diseases; for a better review, see [177]. Besides, it serves as an inhibitor of lipoprotein oxidation and regulates intracellular glutathione redox balance. In vitro studies showed that apoA-IV probably protects against atherosclerosis by promoting several steps in the reverse cholesterol transport pathway. Precisely, apoA-IV activates LCAT and modulates the activation of LPL as well as the protein-mediated transfer of cholesteryl esters from HDL-C to LDL-C.

Cross-sectional studies have shown an inverse relationship between plasma apoA-IV levels and the presence of coronary artery disease in the general population as well as among patients with CKD [145]. Interestingly, apoA-IV plasma levels were found to be already increased when GFR was still within the normal ranges. Moreover, in dialysis patients, apoA-IV levels were twice as high as in the general population [145]. Furthermore, during a prospective 7-year follow-up study, Boes et al. found that increased plasma apoA-IV concentrations predict the progression of primary nondiabetic CKD, independently of baseline GFR. These findings were unexpected, given the physiologic functions in reverse cholesterol transport and the antioxidative properties of apoA-IV [178]. However, this does not necessarily suggest that apoA-IV per se is pathogenic. It is conceivable that apoA-IV reflects the catabolism of apoA-IV in the kidney, which is not fully reflected by the changes in GFR. ESRD is known to influence the serum apoA-IV concentration, especially in HD patients, where there is a marked increase in apoA-IV levels.

Other observations among nephrotic patients suggested that the human kidney is involved in apoA-IV metabolism. This hypothesis is further supported by the presence of apoA-IV in kidney tubular cells.

Characteristics of lipid disorders with an indication of the trends in changes in selected parameters of lipid metabolism at different stages of CKD and the HD and peritoneal dialysis (PD) groups [174] are presented in Table 2.

### 6.3. Disordered NO Metabolism

One of the critical linkages between oxidative stress and atherosclerosis is disordered NO metabolism found in CKD. ROS affect the progression of atherosclerosis by reducing the bioavailability of NO. O_2_^•−^ reacts very rapidly with NO, resulting in the formation of ONOO^−^ responsible for nitration and nitrosylation of amino acid residues. The nitration of SOD in the Tyr-34 position, which occurs due to inflammation, leads to loss of its enzymatic activity [143]. Besides, its protective role is significantly reduced because the reaction of O_2_^•−^ dismutation proceeds more slowly than the reaction of O_2_^•−^ with NO [134]. NO deficiency results in disorders of vascular tone regulation, and VSMC proliferation also promotes platelet activation and aggregation and leukocyte adhesion to endothelium [179].

In addition, the nitration of actin leads to dysfunction of the cellular cytoskeleton, and the nitration of prostacyclin synthase (PGI2) at the Tyr-430 position results in vasoconstriction and increased platelet aggregation [143].

Another essential aspect regarding NO is related to asymmetric dimethylarginine (ADMA), which is predictive of increased mortality and CVD. ADMA induces endothelial dysfunction through a competitive inhibition of the eNOS substrate L-arginine [180,181,182]. The impact of ADMA on NOS is not only limited to competition with L-arginine for binding in the active center. Several studies suggest that there is also direct inhibition of enzyme activity. A potential handle point is the phosphorylation of NOS at the Ser1177 position. ADMA, affecting the cascade of kinases MAPK/ extracellular-signal-regulated kinase (ERK) pathway, inhibits phosphorylation, which in turn prevents the recruitment and activation of NOS [183]. In CKD, ADMA concentration is increased, not only due to decreasing renal clearance and protein catabolism but also because ADMA synthesis and degradation are affected [181]. Post-translational methylation reactions of arginine residues catalyse protein methyltransferases (PRMTs). In turn, ADMA degradation is performed by type 2 dimethylarginine dimethylaminohydrolase (DDAH). ROS act similarly to metabolic acidosis here, so they increase the expression of the PRMT type 1 gene and reduce the expression of the DDAH gene. As a result, an increase in the concentration of ADMA, which inhibits the formation of NO, is observed [184]. The increase in the concentration of oxidative stress markers, such as MDA, ox-LDL, and the increase in ADMA concentration correlates with the degree of endothelial damage in the course of CKD [184,185,186].

### 6.4. Uremic Toxins as Markers of Atherosclerotic Organ Damage

Uremic toxins, one of the non-classical cardiovascular risk factors in patients with CKD, are traditionally categorized based on the physicochemical characteristics affecting their clearance during dialysis. These contained low water-soluble molecules (molecular weight < 500 Da) such as ADMA, urea, uric acid, polyamines; larger middle molecules (molecular weight > 500 Da) such as fibroblast growth factor 23 (FGF-23), parathyroid hormone (PTH), TNF-α, ghrelin, leptin, IL-8, IL-1; and protein-bound molecules such as AGEs, AOPPs, products of the kynurenine pathway and the indole pathway. Most of the middle molecules are generated endogenously, contrary to the small water-soluble compounds and the protein-bound solutes, which, to a large extent, are intestinal metabolites of nutrition components. Excessive uremic toxins are produced as a result of gut microbiota alteration, including indoxyl sulfate, *p*-cresyl sulfate, and trimethylamine-N-oxide, which are all implicated in the variant processes of kidney diseases development; for a better review, see [187].

The major metabolic pathways involved in the production of uremic toxins include (1) gastrointestinal ingestion and direct, unmodified absorption through the gut wall into the systemic circulation (e.g., AGE); (2) gastrointestinal uptake of a precursor (e.g., an amino acid) that is converted by the intestinal microflora to another precursor (e.g., indole); once absorbed through the gut wall and portal vein, this precursor is further conjugated by the liver (e.g., indoxyl sulfate) before being transferred to the systemic circulation; (3) gastrointestinal absorption of a precursor (e.g., amino acid) that is converted by the intestinal microflora to another precursor (e.g., indole); the precursor is conjugated upon absorption through the gut wall (e.g., indoxyl sulfate) and then enters the systemic circulation through the liver without further modification; (4) endogenous production without the involvement of the gastrointestinal tract (e.g., β2-microglobulin) [188].

In patients with CKD, the accumulation of products of both the kynurenine pathway and the indole pathway correlates with markers of atherosclerotic organ damage, such as the presence of coronary disease [130], the carotid intima-media thickness (IMT) [189], ankle–brachial index [190], and echocardiographic markers of left ventricular diastolic failure [190]. Activation of the aromatic hydrocarbon receptor (AhR) by tryptophan metabolites has been proven to promote the osteoblastic transformation of VSMC, increase the expression of adhesion molecules (VCAM-1, ICAM-1), as well as disrupt endothelial cell function and endothelial nitric oxide production [132,191]. Oxidative stress also affects the accumulation of protein-related uremic toxins.

Several uremic metabolites and their precursors exert immunomodulatory effects. On the gut side, p-cresol, trimethylamine N-oxide (TMAO), and H_2_S can influence the structure and function of the intestinal barrier and thus stimulate immune cells and contribute to inflammation. Little is known so far about the role of sulfur compounds in the intestines and H_2_S, in particular in the context of CKD where there is profound dysregulation of patients metabolism [192]. Some of the toxins, for example, indole-3-acetic acid (IAA) are reported to predict mortality and cardiovascular events in CKD patients. Selected uremic toxins with their impact on CKD and relation to oxidative stress are presented in Table 3.

**Figure 3:**
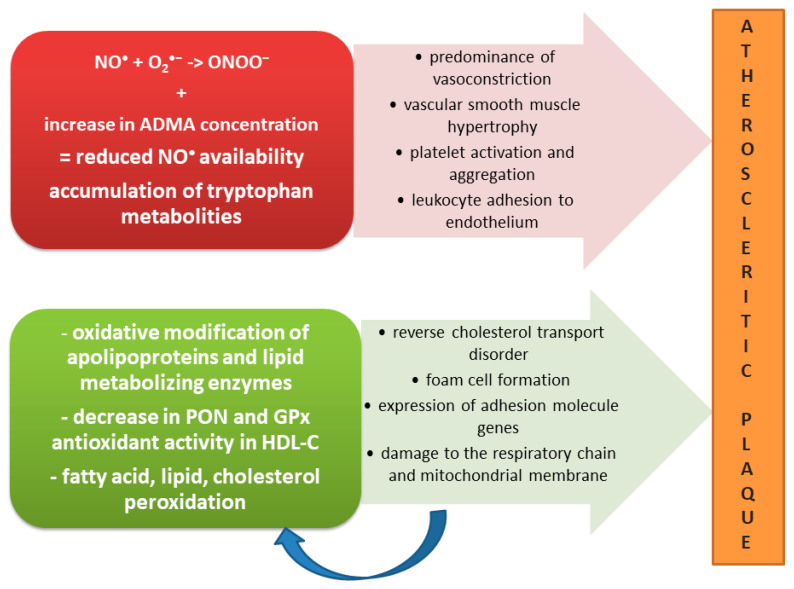
The influence of oxidative stress on the development of atherosclerotic changes in CKD.

The scheme of the influence of oxidative stress on the development of atherosclerotic changes in CKD is presented in Figure 3.

Under oxidative stress, the metabolism of nitric oxide (NO) is disturbed. On the one hand, chronic inflammation stimulates the expression of the inducible NOS gene. On the other hand, metabolic acidosis and the increased presence of ROS cause an increase in ADMA synthesis, which inhibits NO formation. The superoxide anion (O_2_^•−^) also contributes to the decline in NO availability, as it reacts violently with NO to form ONOO^−^, which is responsible for post-translational protein modifications. NO deficiency leads to the advantage of vasoconstriction over vasodilation, vascular smooth muscle cell hyperplasia, the activation and aggregation of platelets, and the increased adhesion of leukocytes to the endothelium. At the same time, the attack of ROS leads to the modification of apolipoproteins by carbamylation, nitration, and glycation, which disturbs the circulation of cholesterol in the body. The lipid components of lipoproteins are also oxidized, which results in the synthesis of such compounds as 4-hydroxy-2-alkenes responsible for damage to the respiratory chain and mitochondrial membrane, oxysterols promoting apoptosis of monocytes/macrophages, VSMC and endothelial cells, and oxidized phospholipids (ox-PL), which increase the expression of adhesion molecules and the activity of NADPH oxidase. ROS also affect the activity of antioxidant enzymes that are components of HDL: PON and GPx. All of these factors contribute to vessel wall damage, foam cell formation, and plaque formation in patients with chronic kidney disease.

## 7. Disorders in Calcium–Phosphate Balance in CKD

CKD causes changes in mineral metabolism that worsen as the disease progresses. Firstly, as GFR decreases, the kidneys lose their ability to excrete phosphorus. Phosphate retention occurs even though serum phosphorus levels are often kept in the routine laboratory range until the relatively late CKD stage. Unlike PTH, an increase in serum phosphorus levels does not become apparent until GFR approaches 30 mL/min/1.73 m^2^. The delayed onset of hyperphosphatemia is probably due to the anti-regulatory effects of FGF-23 and PTH.

Increasing levels of FGF-23 and PTH stimulate phosphorus excretion in urine, which counteracts phosphorus retention. Hyperphosphatemia disrupts the production of calcitriol by reducing the expression of the renal 1α-hydroxylase enzyme, ultimately leading to vitamin D resistance and hypocalcemia, which leads to further abnormalities in the parathyroid glands, bones, and the cardiovascular system.

### 7.1. Vitamin D

Vitamin D is a critical hydrophobic anti-oxidant, whose activity and distribution in the body is disturbed in patients with CKD. There is a growing body of evidence supporting the importance of vitamin D in many vital non-skeletal biological processes, such as endothelial function, RAA system regulation, redox balance, and innate and adaptive immunity. These factors are known as the non-classical effects of vitamin D [204].

Vitamin D deficiency is an element of complex disorders of calcium and phosphate metabolism, in which both oxidative stress and chronic inflammation are involved. Cholecalciferol (vitamin D3) is produced in the skin from 7-dehydrocholesterol by UV. It is also supplied with the diet (vitamin D3 and ergocalciferol (vitamin D2)). Next, from the gastrointestinal tract, it is transported through the vitamin D binding protein (VDBP) to the liver, where they undergo enzymatic 25-hydroxylation. In the kidneys, the 1α-hydroxylation stage occurs, which leads to the formation of the metabolically active form of vitamin D. Both cholecalciferol and ergocalciferol, as well as an active metabolite of vitamin D3—the calcitriol (1,25-hydroxy-cholecalciferol)—play a crucial role in defense against ROS [205,206]. As lipophilic compounds, they bind to membrane phospholipids and protect them against peroxidation, and their antioxidant activity exceeds that of other hydrophobic compounds, such as α-tocopherol or melatonin [207]. There are also many reports documenting the effect of calcitriol on the increase of SOD activity [208], increasing the expression of the glutathione reductase gene [209], and the impact on the synthesis of ROS in the cell by binding to the vitamin D nuclear receptor (VDR) [210].

The impact of calcitriol on the pathway of Nrf2 is also considered. Under normal conditions, the Nrf2 transcription factor is present in the cytosol in a complex with the Keap1 (key postinduction repressor of Nrf2 repressor). The reaction of ROS with Keap1 changes its conformation and leads to the release of Nrf2 from the complex. A similar effect is also given by the phosphorylation of Nrf2 with the participation of signaling kinases in the cell (PKC, MAPK). Under the influence of the released Nrf2, the genes encoding enzymes (catalase, glutathione S-reductase, thioredoxin reductase) responsible for fighting ROS, are transcribed [211,212].

Two pathways are considered to show the impact of calcitriol on Nrf2: increasing the expression of Nrf2 transcription factor and decreasing the expression of Keap1 [213,214]. Calcitriol deficiency in the course of CKD results from a decrease in the activity of 1-α-hydroxylase, which is an enzyme that is found mainly in the cells of the renal tubular epithelium [215,216]. Urinary loss of VDBP and dietary deficiency affect peripheral 1-α-hydroxylation [217,218].

Vitamin D metabolism disorders are accompanied by other calcium phosphate abnormalities that underlie the pathogenesis of CKD and are associated with increased oxidative stress.

Vitamin D deficiency (<20 ng/mL) and insufficiency (20–29 ng/mL) are common among patients with CKD or undergoing dialysis. In CKD, impaired kidney hydroxyvitamin D (25(OH) D), the primary circulating form of vitamin D uptake, remains the leading cause of 1,25(OH)_2_ D deficiency, as the metabolic clearance rate of calcitriol appears to be unchanged. Apart from the direct influence of high 25(OH) D levels, the local osteoblastic conversion of 25(OH) D to 1,25(OH)_2_ D seems to be an essential positive regulator of FGF-23 production, especially in uremia. Causes and risk factors for 25(OH) D deficiency or insufficiency observed in CKD and dialysis patients are involved, among them, one can consider: (1) age, female sex, adiposity, (2) proteinuria, (3) low physical activity, (4) peritoneal dialysis, (5) diabetes mellitus, (6) reduced VDR, reduced skin synthesis of vitamin D; (7) impaired 25(OH) D tubular reabsorption, (8) calcineurin inhibitor prescription, and (9) reduction of the liver CYP450 isoform in secondary hyperparathyroidism [219]. Notably, there are many significant consequences of these disturbances, observed especially among dialyzed patients, such as (1) muscles weakness, (2) low bone mineral density, (3) secondary hyperparathyroidism and increase in bone turnover markers, (4) metabolic syndrome, obesity, insulin resistance, (5) left ventricular hypertrophy and atherosclerosis, (6) vascular calcification and arterial stiffness, (7) progression of kidney function, (8) cognitive impairment, and (9) increased mortality [204].

Wolf et al. studied the associations between early mortality incidents and vitamin D levels among HD patients. These researchers performed a cross-sectional analysis of 825 consecutive incident HD patients across 569 HD centers in 37 states in the USA. Patients who died within 90 days of initiating dialysis were compared with those who survived for at least 90 days. Individuals presenting with 25(OH) D < 10 ng/mL were at significantly increased risk of all-cause and cardiovascular mortality compared to subjects with 25(OH) D > 30 ng/mL, while patients with 25(OH) D levels 10–30 ng/mL showed mixed results after multivariate adjustments [220].

### 7.2. Parathormon (PTH)

PTH is an 84-amino acid polypeptide secreted in response to hypocalcemia via calcium-sensing receptors (CaSR) present on the surface of the parathyroid glands. Under physiological conditions, the expression of the PTH gene is inhibited by calcitriol, which simultaneously stimulates the expression of the CaSR gene [221].

In CKD, calcitriol deficiency and a decrease in the number of VDR cause an increase in PTH secretion. It leads to the development of secondary hyperparathyroidism, in which high levels of PTH caused by chronic hypocalcemia and calcitriol deficiency accompany hyperphosphatemia [216,221]. Due to the high importance of hyperparathyroidism in the pathophysiology of CKD and associated calcium–phosphate complications, PTH is referred to as uremic toxin [221]. The persistence of calcium phosphate disorders leads to a polyclonal proliferation of parathyroid glands with a decrease in the expression of VDR and CaSR receptors, resulting in the autonomization of parathyroid function, which is referred to as tertiary hyperthyroidism. This disturbance appears in patients with ESRD and is characterized by extremely high levels of PTH, as well as hypercalcemia, hyperphosphatemia, and elevated levels of osteolysis and osteogenesis indicators [221,222]. Hyperparathyroidism, together with calcitriol deficiency, has a negative effect on the skeletal system. Due to the decrease in calcitriol concentration, the proper mineralization of osteoid is impossible, and calcitriol deficiency interferes with the proper effect of PTH on osteoblasts and osteoclasts [215,222].

Bone metabolism disorders accompanied by renal osteodystrophy and soft tissue calcification develop, including medial artery calcification, which is the most typical for CKD. Calcification is a complex and tightly regulated process involving inhibitors, inducers, and cell differentiation processes. In addition, it has become apparent that cardiovascular symptoms of calcification predict patient outcomes in general populations, but in particular in patients with CKD. Vascular calcification is associated with increased morbidity and mortality. However, its multifactorial mechanism is not fully understood.

Oxidative stress was found to oxidize PTH, mainly methionine residues at positions 8 and 18. Receptors do not recognize such modified molecule in tissues, and a loss of biological activity of PTH may contribute to calcium phosphate disorders in ESRD [223]. However, in most studies published so far, it has not been proven that PTH concentration directly correlates with the increase in the level of oxidative stress markers [224,225]. An exception is a study by Nakatani et al., in which the concentration of PTH correlated with the concentration of the oxidized form of albumin in which the thiol group Cys-34 formed a disulfide bond. However, no similar correlation was found for FGF-23 concentration [226].

On the other hand, in vitro studies revealed that under the influence of elevated PTH levels, elevated mitochondrial ROS production and endothelial cell dysfunction occurred. However, it should be noted that the intensity of ROS production under the influence of PTH is not as high as in hypoxia-induced oxidative stress. It is suggested that under the influence of PTH, there is no direct cell damage and apoptosis as a result of leakage of ROS from mitochondria, but rather the disruption of signaling pathways due to the oxidative modification of proteins such as bradykinin receptors and growth factors [200].

Besides, in the study by Lishmanov et al., the PTH level in CKD patients (stages 3 and 4) was associated with an increased incidence of cardiovascular events independent of calcium–phosphorous level, kidney function, previous history of vascular disease, and serum 25(OH) D levels [227]. Researchers pointed to the impact of PTH on vascular endothelial function contributing to increased vascular tone and stiffness. Notably, PTH may play a role in the calcification of atherosclerotic lesions. In an experimental study evolving the infusion of PTH, both rats with uremia and those with normal renal function developed intense medial aortic calcification, and some animals also developed coronary calcification. It is known that AGE and IL-6 are associated with atherosclerosis and inflammation, and PTH has been shown to stimulate the expression of AGE and IL-6 mRNA and expression of AGE receptor proteins in endothelial cells. Percovic et al., in their in vitro study, found that PTH increases the production and reorganization of collagen by VSMCs. The researchers suggested that it seems that a more aggressive control of hyperparathyroidism in patients with renal failure may help to reduce the burden of cardiovascular disease in this patient population [228].

### 7.3. Klotho Protein

It has been observed that even only a slight decrease in GFR results in a downregulation of renal α-klotho (klotho). The klotho protein exists in several forms, including the full-length transmembrane form (mKL), a soluble circulating form (soluble klotho (sKL)), and a secreted truncated form. In the transmembrane form, it occurs in the epithelial cells of the renal tubules and collecting coils, where it is highly expressed compared to other tissues [229]. Under the influence of the secreted form of klotho, the epithelial calcium (Ca^2+^) channels TRPV5 retain on the surface of renal tubule cells, which allows increased calcium reabsorption in the nephron [230]. The mKL form acts as a co-receptor for FGF-23, which is secreted by osteocytes in response to an increase in calcitriol concentration. The effect of FGF-23 on renal tubular epithelial cells is to reduce the expression of Na-Pi IIa and IIc transporters, which increases urinary phosphate excretion [231]. Klotho is associated with oxidative stress via the insulin/insulin-like growth factor 1 (IGF-1) pathway. The binding of insulin/IGF-1 receptor with ligand causes the activation of phosphatidylinositol 3-kinase (PI3K), phosphorylation of phosphatidylinositol-4,5-bisphosphate (PIP2) to phosphatidylinositol-3,4,5-trisphosphate (PIP3), activation of protein kinase 1 from phosphatidylinositol (PDK1), and phosphorylation and activation of protein kinase B (Akt/PKB). Active Akt/PKB performs the phosphorylation of numerous proteins in various cell compartments, including forkhead transcription factors of the O class (FOXOs). Recent studies have demonstrated that FOXOs play critical roles in a wide variety of cellular processes; see [232]. FOXOs transcriptionally activate or inhibit downstream target genes, thereby playing a significant role in proliferation, apoptosis, autophagy, metabolism, inflammation, differentiation, and stress resistance. The FOXO transcription factor loses its function after phosphorylation and remains in the cytoplasm instead of being shifted to the cell nucleus. However, in the presence of klotho, the activity of the insulin/IGF-1 pathway is inhibited, and there is no phosphorylation of FOXO, which is transported to the nucleus. As a result, the nuclear FOXO directly binds to the SOD-2 promoter and upregulates its expression, thereby facilitating ROS removal and providing resistance to oxidative stress [229,233].

Studies have revealed the pleiotropic effect of klotho on bone, the cardiovascular system, and even as a tumour-suppressor molecule [187,234]. Some researchers suggest that (sKL) binds to multiple Wnt ligands, suppressing various gene transcriptions. Increasing klotho regulation stops Wnt activation, which reduces extracellular matrix deposition and cytokine transcription [234]. It seems that klotho deficiency may contribute to the cardiac hypertrophy observed in CKD (stages G3a-b and G4). For CRS type 2, tests in mice with heart failure revealed potential mechanisms linking cardiac and renal dysfunction. In these mice, an increase in cardiac remodeling was associated with a significant reduction in klotho and activation of the Wnt/β-catenin system and RAA. All heart changes worsened due to renal-dependent klotho deficiency. The presence of sKL partially inhibited Wnt/β-catenin signaling, which in turn promoted the downregulation of cardiac fibronectin and α smooth muscle actin. Thus, Wnt/β-catenin activity in the heart is accompanied by kidney disease. At the same time, klotho deficiency resulting from renal failure worsened cardiac remodeling. The involvement of Wnt signalling activation in CVD pathogenesis has been extensively studied [235]. A relationship has been found between Wnt signaling activation and atherosclerosis. Between the severity of the atherosclerotic lesion and the level of Wnt in the serum, a positive correlation was revealed. In addition, Wnt staining was detected in internal macrophage accumulation areas in atherosclerotic lesions in both apolipoprotein-deficient mice and in post-endarterectomy human samples. Christman et al. discovered that ox-LDL could induce Wnt expression. In turn, other authors showed that increased levels of ox-LDL induce a decrease in renal expression of klotho [235]. A recent study conducted in patients treated by HD showed that higher serum FGF-23 levels and lower levels of sKL and sclerostin (endogenous Wnt inhibitor) were associated with chronic inflammation, malnutrition, and secondary hyperparathyroidism, and they may be considered as prognostic factors for CVD and its complications, such as left ventricular hypertrophy, acute coronary syndrome, or cardiac arrhythmias [236]. On the other hand, data on the relationship between klotho and kidney function are divergent. Some studies have shown that a decrease in serum klotho correlates with a reduction in renal function; nevertheless, in other studies, such a relationship has not been confirmed [237,238]. Similar controversies concern the link between klotho and both PTH and FGF-23 [187]. Other studies indicate the key role of klotho concentration in urine, not in serum, as a prognostic parameter in CKD [96]. Difficulties in assessing klotho impact arise from both its multidirectional effects and reservations about differences in its transmembrane and secretory forms.

On the other hand, Yamamoto et al. have proved in animal models that an increase in serum klotho concentration results in a decrease in urine concentration of 8-hydroxy-2-deoxyguanosine (8-OH-dG), which is a key marker of oxidative DNA damage [233]. In turn, in vitro studies, under the influence of paraquat, which is responsible for the production of O_2_^•−^, the klotho protein inhibited lipid peroxidation [239]. Raesi et al. found that among rats receiving ciclosporin, the serum concentration of klotho correlated inversely with the concentration of 8-hydroxy-2-deoxyguanosine (8-OH-dG) and MDA [240]. The study by Oh et al. that included 78 patients on PD revealed the inverse correlation between serum klotho protein and 8-isoprostanoid concentration [241].

### 7.4. Disturbances in Calcium–Phosphate in CKD

Disorders of vitamin D, PTH, and klotho protein in ESRD lead to the development of distant complications, such as renal osteodystrophy, soft-tissue calcification, and calciphylaxis (calcific uremic arteriolopathy). Reduced calcitriol concentrations reduce the absorption of calcium and phosphate in the gastrointestinal tract and at the same time stimulate the parathyroid glands to secrete PTH, which leads to the development of hyperparathyroidism. Not only is calcitriol deficiency found in CKD patients, but a decrease in klotho protein concentration and an increase in FGF-23 concentration are found as well. It has been observed that in the early stages of CKD when hyperphosphatemia and hyperparathyroidism have not yet developed, there are elevated levels of FGF-23 [242,243]. Most likely, in the early stages of CKD, increased FGF-23 secretion allows normophosphatemia to be maintained, despite the increasing loss of renal excretory function and reduced expression of klotho protein in renal tubular cells. However, at the same time, high levels of FGF-23 cause calcitriol deficiency by inhibiting its synthesis in the kidneys. PTH secretion disorders are also associated with increasing renal–bone axis disorders. Depending on other existing disorders, numerous disturbances in calcium–phosphate homeostasis occur in CKD; see [244,245].

The critical issue is the relationship between oxidative stress, calcium phosphate disorders, and medial artery calcification that is characteristic of CKD. Under the influence of hyperphosphatemia, the increased influx of phosphates into VSMC, through the membrane type III Na(+)-dependent phosphate (NaPi) transporter Pit-1 isoform, causes osteoblastic transformation and the synthesis of proteins stimulating calcification [246]. Hyperphosphatemia also prevents the proper maturation of macrophages into osteoclast-like cells that are responsible for osteoid resorption [247]. In turn, hypercalcemia inhibits the synthesis of calcification inhibitors such as fetuin-A [248]. Fetuin-A is an inflammatory Ca-linked glycoprotein and a prototype of a systemic calcification inhibitor. The emerging role of fetuin-A deficiency as a risk factor in dialysis patients has been documented in cross-sectional studies showing a significant correlation with total mortality and cardiovascular mortality [249]. The coexistence of fetuin-A deficiency, together with atherosclerotic vascular damage and hyperphosphatemia, leads to a significant increase in vascular calcification in CKD [250].

**Figure 4:**
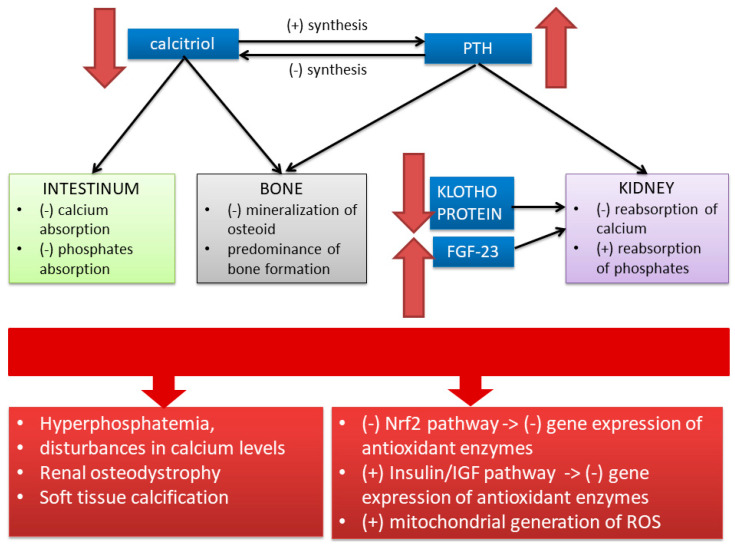
The effect of oxidative stress on calcium and phosphate disorders in CKD.

Besides, hypercalcemia, as well as hyperphosphatemia, synergistically potentiate VSMC apoptosis [251]. An important role is also played by deregulation of factors controlling calcium and phosphate metabolism: calcitriol, PTH, and klotho protein. Secondary hyperparathyroidism accelerates bone circulation with the release of large amounts of calcium and phosphate into the plasma. In turn, klotho protein deficiency stimulates the Na-Pi III Pit-1 counter transporter, which promotes VSMC osteoblastic transformation. However, the relationship between FGF-23 and calcification of the medial artery calcification is unclear. Perhaps the effect of FGF-23 is due to interaction with other factors such as PTH and Klotho protein, and not directly to the process of calcification [251]. The scheme representing the effect of oxidative stress on calcium and phosphate disorders in CKD is presented in Figure 4.

As CKD progresses, there is an increasing deficiency of calcitriol and an increase in FGF-23 concentration, which is accompanied by abnormalities of secretion and synthesis of PTH and Klotho protein. As a result, systemic disorders of calcium and phosphate metabolism develop, including disorders of intestinal absorption, impaired excretion in the kidneys, and disorders of proper bone tissue formation. In end-stage renal disease, hyperphosphatemia accompanied by hyper- or hypocalcemia is observed. Secondary hyperparathyroidism is associated with renal osteodystrophy with increased bone turnover, while deficient PTH levels are associated with adynamic bone disease. In patients with end-stage renal failure, soft tissues may also be calcified. Clinical indicators of calcium–phosphate disturbances are accompanied by redoxy imbalance. Calcitriol increases the expression of the Nrf2 transcription factor gene and inhibits the expression of the Keap1 repressor protein gene. Therefore, calcitriol deficiency has an inhibitory effect on the Nrf2 pathway, which results in the decreased expression of genes of antioxidant enzymes (catalase, glutathione S-reductase, thioredoxin reductase). In turn, the Klotho protein inhibits the insulin/IGF-1 pathway, which corresponds, among others, to phosphorylation and inactivation of the transcription factor FOXO. The Klotho protein deficiency leads to increased activity of the insulin/IGF-1 pathway, as well as increased phosphorylation and inactivation of the FOX transcription factor, and as a result to reduced expression of the gene for the key antioxidant enzyme: mitochondrial superoxide dismutase (SOD-2). On the other hand, high PTH concentration is associated with increased mitochondrial ROS production and the oxidative modification of proteins.

## 8. Anemia in CKD

The coexistence of CKD, heart failure, and anemia is often found in clinical practice. While these conditions are individually associated with significant morbidity and mortality, the presence of the triad promises an even worse prognosis. Anemia is common among patients with CKD and heart failure, indicating that its presence may serve to explain poor results in these populations. It is well known that erythropoiesis is impaired as kidney function deteriorates, which is associated with an increased risk of mortality and adverse CVD events. However, trials of erythropoietin-stimulating agents (ESA) have had different results at cardiovascular end points. Evidence is now emerging to suggest that treating erythropoietin (Epo) deficiency alone may be as important or more important than achieving normal hemoglobin levels [252].

The causes of anemia in CKD are complex. They relate to erythropoiesis disorder, increased erythrocyte destruction, and increased incidence of hemorrhagic incidents.

### 8.1. Reduce in Erythrocytes Lifespan

One of the reasons for reducing the lifespan of erythrocytes from 120 to 80 days is oxidative stress [78,253]. Recent evidence suggests that oxidative stress is an essential factor accelerating erythrocyte loss; thus, it is one of the critical mechanisms of anemia in CKD. Oxidative stress can injure erythrocytes and finally may lead to their suicide or eryptosis (apoptosis or programmed death of erythrocytes). Oxidative stress activates Ca^2+^ non-selective cation channels in the cell membrane, thus stimulating Ca^2+^ entry; then, it activates Ca^2+^-sensitive K^+^ channels, leading to K^+^ exit, hyperpolarization, followed by Cl^−^ exit, and eventually cell contraction due to loss of KCl and driven water osmotically [254]. Besides, ROS may modify skeletal membrane proteins of erythrocytes, especially those located peripherally, such as ankyrin and spectrin, leading to erythrocytes’ increased susceptibility to hemolysis [255]. Even blood loss resulting from increased erythrocytes destruction is not significant but challenging to compensate for due to abnormalities in the erythropoiesis process. On the other hand, convincing evidence suggests that anemia in CKD is mainly the result of accelerated suicide of erythrocytes [256]. Apart from the eryptosis, the disruption of hematopoietic processes is present in CKD. It results from both bone marrow suppression by uremic toxins, such as PTH and polyamines, and from Epo deficiency. The decrease in Epo is the result of damage to type I periarticular cells in the interstitial cortex, and its severity is related to anemic progression. Since Epo not only affects hematopoietic cells but also has a systemic effect, disturbances in its synthesis and function result in more severe complications than anemia itself. Among others, Epo stimulates the expression of antioxidant enzyme genes by affecting the NF-κB nuclear transcription factor pathway and the Nrf-2 factor pathway [74], and it also reduces ROS production by restoring eNOS [74].

### 8.2. Epo Deficiency in CKD

A deficiency of Epo causes further disturbance of the redox balance in the organism. In addition to interstitial cell damage, other mechanisms associated with increased oxidative stress and chronic inflammation are also observed. Circulating Epo may be carbamylated as a result of the oxidative action of isocyanate, which is formed in the process of urea dissociation. In this way, there is a loss of function because carbamylated Epo is not recognized by the receptor on the cell surface of the erythropoietic line [126]. In approximately 10% of patients, the administration of ESA does not give the expected improvement in CKD. The phenomenon of resistance to ESA results from both an increased oxidative stress and chronic inflammation, which prevents the proper functioning of the cells of the erythropoietic line [257,258,259]. Patients with known ESA resistance showed increased levels of oxidative stress markers such as MDA and oxidized DNA, with reduced levels of antioxidant enzymes (erythrocyte SOD and leukocyte CAT) [259]. Proinflammatory cytokines, to increase the synthesis of innate immune effector cells and inhibit steady-state erythropoiesis, alter bone marrow hematopoiesis. They affect the metabolism and activity of Epo by inhibiting its production in the kidneys (IL-1, TNF-α) and reducing the expression of the Epo receptor (EpoR) gene in the bone marrow (INF-γ) [126]. TNF-α inhibits erythropoiesis through direct and indirect effects. Indirectly, TNFα activates NF-κB and GATA binding protein 2 (GATA-2) transcription factors that have also been reported to be involved in inhibiting Epo production by blocking hypoxia-inducible transcription factor 1 alpha (HIF-1α) in vitro. In turn, low levels of Epo reduce EpoR-mediated signaling pathways, resulting in a decrease in GATA-1 (erythroid transcription factor) levels, and consequently, a possible deregulation of EpoR expression. TNF-α has also been shown to have a direct effect via its receptors, leading to activation of the NF-κB canonical pathway (p50/p65), which inhibits the expression of erythrocyte-specific genes. TNFα has also been reported to activate GATA-2, whose over-expression inhibits erythropoiesis and promotes megakaryopoiesis. Thus, the action of TNF-α causes a reduction in the expression of Epo genes and hemoglobin production [260]. Moreover, secondary hyperparathyroidism was discovered to have a significant cause of ESA hyporesponsiveness in CKD. The pathogenesis of anemia in secondary hyperparathyroidism is still not so distinct. However, it can be attributed to the reduced production of red blood cells (RBCs) from calcitriol deficiency or the inhibition of erythropoiesis by PTH, which is associated with bone marrow fibrosis and the disruption of red blood cell survival [261].

### 8.3. Iron Disturbances in CKD

Iron disorders are other factors that promote anemia in patients with CKD. In the course of CKD, patients develop an inflammatory block of iron metabolism—deregulation of the hepcidin–ferroportin axis—which is manifested by characteristic laboratory changes (low iron transferrin saturation, high ferritin concentration) and resistance to iron supplementation. Ferroportin, a protein found on the surface of duodenal enterocytes, hepatocytes, and macrophages, is responsible for transporting iron from the cells to the extracellular space. In turn, hepcidin is produced in the liver and secreted into plasma. Hepcidin binds to ferroportin, leading to its degradation, followed by a decrease in cellular iron export. Hepcidin also reduces the expression of the transferrin gene, which is a critical iron-transporting protein [262]. In the course of CKD, hepcidin levels increase due to a decrease in renal clearance and an increase in liver synthesis. Proinflammatory cytokines, especially IL-6, via the signal transducer and activator of the transcription 3 (STAT-3) signaling pathway, are an additional factor stimulating expression of the hepcidin gene [262,263]. As a result, the gastrointestinal tract impairs iron absorption, and iron present in the body is trapped in the cells of the reticuloendothelial system [263,264]. However, the direct impact of oxidative stress on hepcidin synthesis is debatable, since the relationship between oxidative stress markers and hepcidin concentration has not been proven [265]. Besides, lipid peroxidation products, such as 4-hydroxy-2-nonenal (4-HNE), were found to induce the expression of cyclooxygenase 2 (COX-2), which leads to the synthesis of proinflammatory eicosanoids and the intensification of anemia by enhancing chronic inflammation [266].

Due to iron disorders, resulting in reduced iron absorption from the gastrointestinal tract, parenteral supplementation is required in some patients. It is especially crucial for patients on the HD program, where the renal replacement technique itself facilitates the intravenous administration of a solution of iron compounds. It ensures the adequate availability of iron ions for cells of the erythropoietic line in the bone marrow, but at the same time, it intensifies redox disorders in the body. Transition metal ions such as iron, copper, manganese, and nickel are the catalyst for Fenton’s reaction. The Fenton reaction, in the presence of a transition metal ion, creates from hydrogen H_2_O_2_ a highly reactive HO^•^, and the next stage regenerates the oxidized metal ion with the participation of radical O_2_^•−^ [267]. Both the Fenton reaction and the regeneration reaction occur spontaneously and at a high rate, which enables the generation of significant amounts of the highly reactive HO^•^. Under physiological conditions, most iron ions are bound to proteins, which prevents Fenton reaction. CKD disrupts intra-systemic hemostasis, which results in an increased availability of iron ions in the plasma.

Additional intravenous iron supplementation causes among CKD patients a significant increase in oxidative stress markers: lipid peroxidation products, oxidized DNA, and AOPPs [268,269,270].

**Figure 5:**
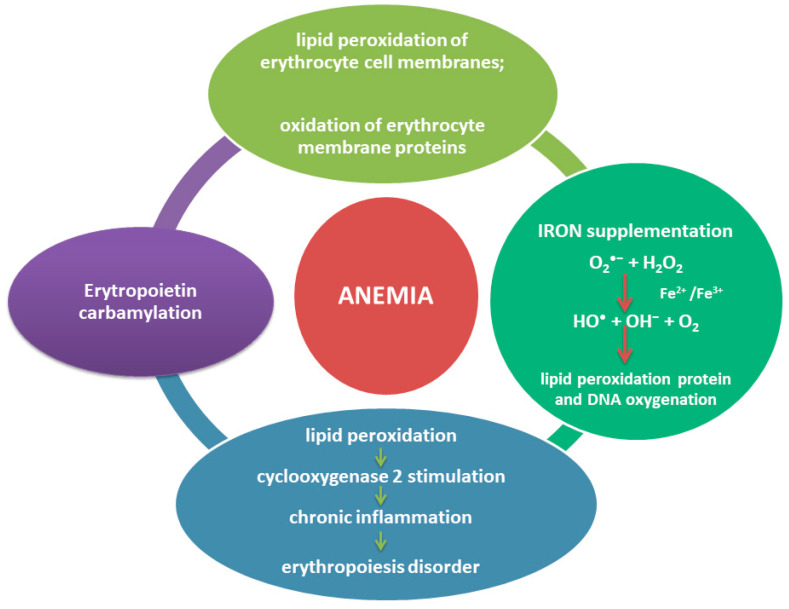
Anemia and oxidative stress in the course of chronic kidney disease.

This effect is less pronounced when a solution containing iron ions is administered continuously or in small portions, which reduces the risk of iron transferrin saturation and the appearance of free iron ions in the plasma [270]. The severity of oxidative stress is also reduced by preparations in which iron forms a complex with sugars, such as sucrose, isomaltose, and carboxymaltose [270,271,272,273]. A scheme presenting the relations between anemia and oxidative stress in the course of CKD is shown in Figure 5.

Oxidative stress, developing in patients with chronic kidney disease, leads to the increased oxidation of lipids and erythrocyte membrane proteins, which significantly shortens their life span. At the same time, bone marrow erythropoiesis is suppressed. Post-translational modifications of erythropoietin by ROS result in a loss of its activity. In turn, lipid peroxidation products stimulate the expression of the cyclooxygenase 2 (COX-2) gene and the synthesis of proinflammatory eicosanoids. Increasing hypercytokinemia inhibits both the synthesis of erythropoietin in type I cells of the renal interstitium and the synthesis of the erythropoietin receptor in bone marrow. On the other hand, intravenous iron supplementation enables the correction of iron deficiency in patients with end-stage renal disease but, on the other hand, it promotes the production of ROS. The free iron ions in the plasma enable the formation of a highly reactive hydroxyl radical (HO^•^), which initiates the oxidation reactions of proteins, lipids, and nucleic acids.

## 9. Cardiovascular Risk in CKD

Cardiovascular events both in environmental studies and in selected populations diagnosed with CVD seem beyond doubt to be strongly related to the level of renal function. In the Framingham Heart Study, the first community study addressing this issue, the link was evident but limited to the male gender only [274]. In addition, in the Atherosclerosis Risk in Communities (ARIC) study, middle-aged men and women with moderate renal insufficiency were found to be at a 38% higher risk of developing atherosclerotic complications than in subjects with normal renal function [275]. Although the National Health and Nutrition Examination Survey (NHANES I) [276] study found no excessive risk in people with renal insufficiency, the NHANES II [277] analysis did. Moreover, a pooled analysis of these four community studies (including the Framingham Heart Study and ARIC) found that moderate renal failure carries a 19% greater risk of cardiovascular complications [278].

It should be underlined that the existence of renal failure in a patient shows a very high-risk situation, especially among patients with pre-existing CVD. In patients with essential hypertension, the relationship between serum creatinine and cardiovascular risk was disclosed to be evident, even within the normal range for serum creatinine. In the Italian study on a group of 1829 patients with hypertension and serum creatinine levels within normal limits, during the 11 years of follow-up, an increase in serum creatinine by 0.23 mg/dl was associated with a 30% higher risk of cardiovascular events [279]. Italian researchers concluded that a serum creatinine value within the reference limits could be a predictor of cardiovascular morbidity in white patients with essential hypertension.

In a retrospective cohort study conducted by the Kaiser Permanente Center, only a small group of patients (approximately 1%) with mild to moderate renal impairment developed ESRD over a 5-year follow-up [280]. However, as many as 19% and 24% of patients with mild and moderate renal failure, respectively, died, mainly from atherosclerotic complications, within the same five years. Thus, the real risk of kidney failure is with the cardiovascular system, not with the kidneys. Go et al. pointed out the independent graded association between lower levels of the estimated GFR and the risks of death, cardiovascular events, and hospitalization. These risks were evident at an estimated GFR < 60 mL/min/1.73 m^2^ and substantially increased with an estimated GFR < 45 mL/min/1.73 m^2^.

It has been estimated the cardiovascular risk in patients who have reached ESRD stage is enormous: five times higher than normal in patients with ESRD aged 85–95 years, and 65 and 500 times higher than normal in those aged 45–54 years and 25–35 years, respectively [281].

The Chronic Renal Insufficiency Cohort (CRIC) study found an inverse correlation between renal function and inflammatory biomarkers (IL-1β, IL-1 receptor antagonist, IL-6, TNF-α, C-reactive protein, and fibrinogen) as well as a direct relationship between kidney disease and albuminuria. It has also been observed that higher levels of acute-phase reagents (C-reactive protein, CRP) and proinflammatory cytokines (especially IL-6) strongly predict cardiovascular morbidity and mortality in patients with CKD [282].

Sun at al. in their post hoc analysis of data from a longitudinal study conducted at the Karolinska University Hospital Huddinge among dialyzed patients found that in addition to age and comorbidities, only IL-6, sVCAM-1, and albumin could, independently of other biomarkers, classify clinical CVD, and only IL-6, WBC, and TNF could, independently of other biomarkers, predict all-cause mortality risk. This study compared the predictive strength of 12 biomarkers analyzed concomitantly in 543 patients with stage 5 CKD. During follow-up for a median of 28 months, there were 149 deaths, 81 of which were caused by CVD [123].

## 10. Conclusions

Oxidative stress accompanied by inflammation (manifested by an increase of inflammatory markers, including cytokines, acute-phase proteins, and adhesion molecules, in which the cells of the innate immune response system are mainly involved) has a significant role in the pathogenesis of CKD. The redox imbalance has a negative effect on all kidney elements, from the renal circulatory system, through the individual components of the glomeruli and renal tubules, to interstitial tissue. The multidirectional effect of ROS results in a disruption of the excretory function on each segment of the nephron, which prevents the maintenance of intra-systemic homeostasis and leads to the accumulation of metabolic products. Renal regulatory mechanisms are also disrupted: tubular glomerular feedback, myogenic reflex in the supplying arteriole, and RAA. It makes it impossible to compensate for water–electrolyte and acid–base disturbances, which continue in the positive feedback mechanism, leading to the further intensification of oxidative stress. As a result of the increase in chronic inflammation and oxidative stress, the progression of CKD to ESRD is observed, with a full spectrum of complications such as malnutrition, calcium phosphate abnormalities, atherosclerosis, and anemia contributing to the acceleration of their CVD. In our review, we identified key factors responsible for disorders occurring in CKD. The complexity of their action is enormous and is still recognized in many studies. It is probably the reason for the numerous overlapping complications, of which cardiovascular problems are crucial, contributing to the increased mortality in CKD.

## Figures and Tables

**Table 1:** The changes in the antioxidative system in chronic kidney disease (CKD), with particular emphasis on glutathione peroxidase (GPx), superoxide dismutase (SOD), and catalase (CAT). HD: hemodialysis, PD: peritoneal dialysis, HV: healthy volunteers.

Parameter	Study Group				
	Non-Dialyzed CKD vs. HV	HD	before HD vs. after HD	PD	Nephrotic Syndrome
**E-GPx**	↑ [44,73] ↓ [68,69,71,74,79] N [58,76,78]	↑ [44] ↓↓ [69,70,74,75,78,79] N [58,68,71,80]	no change [68,70,71,80] ↘ [75]	↓ [74,78] N [58,71,78]	↓ [67,68]
**P-GPx**	↘ [44] ↓ [67,68,69,73,76]	↓↓ [44,58,68,69,80,81,82]	↗ [68,80]	↓ [44,58]	↓ [67] N [68]
**SOD-1**	↑ [38,41,72,73] ↗ [74] ↓ [77] N [27,44,76,78,79]	↑ [41,72,80] ↓ [27,38,74,75,77,78] N [44]	↘ [80]	N [44] ↓ [74,78]	↓ [67]
**CAT**	↘ [74,78]	↑ [69] ↓↓ [74,77,78,79]	↗ [77]	↓↓ [74,78]	

↘ or ↗—gradual decrease or increase along with the CKD progression (in non-dialyzed CKD vs. HV or trend of the changes (in the group before HD vs. after HD); ↑, ↓ or ↓↓—increased or decreased value in comparison to HV; N—within the normal ranges.

**Table 2:** Changes of lipid parameters in non-dialyzed and dialyzed patients with CKD. IDL: intermediate-density lipoprotein, LDL: low-density lipoprotein cholesterol, VLDL: Very low-density lipoprotein cholesterol, HD: hemodialysis, PD: peritoneal dialysis.

Parameter	Trends of Changes in the Course of Deterioration of Kidney Function (CKD Stages from 1 to 5)	HD	PD
Total cholesterol	↗	N or↓	↑
LDL cholesterol	↗	N or ↓	↑
Lp (a)	↑	↑↑	↑
HDL cholesterol	↓	↓	↓
Non–HDL cholesterol (includes cholesterol in LDL, VLDL, IDL, and chylomicron and its remnant)	↗	N or↓	↑
ApoA-I	↘	↓	↓
ApoA-IV	↗	↑	↑
ApoB	↗	N or ↓	↑
Triglycerides	↗	↑	↑↑

N—within the normal ranges; ↑, ↓ or ↑↑—show the tendency in the course of CKD and among dialyzed patients. ↘ or ↗—gradual decrease or increase along with the CKD progression.

**Table 3:** Selected uremic toxins with their impact on CKD and relation to oxidative stress. ADMA: asymmetric dimethylarginine, MPO: myeloperoxidase, AGEs: advanced glycation end products, AOPPs: advanced oxidation protein products, VSMC: vascular smooth muscle cells, CMPF: 3-Carboxy-4-methyl-5-propyl-2-furanpropionate, PTH: parathyroid hormone, FGF-23: fibroblast growth factor 23.

Uremic Toxin	Impact of the Uremic Toxin on Complications Observed in CKD	Relations to Oxidative Stress in CKD	References
**Low Water-Soluble Molecules (Molecular Weight < 500 Da)**			
ADMA	hypertension; atherosclerosis	MPO secretion	[181,193]
Polyamine	anemia; suppression of the immune system coagulation disorders	glutathione metabolism (VSMC)	[194,195]
Carbamylated compounds	kidney damage; anemia; atherosclerosis; calciphylaxis	respiratory burst (neutrophils)	[196]
Uric acid	kidney damage; chronic inflammation; hypertension; atherosclerosis	ROS release from mitochondria	[194,197]
**Protein-Bound Molecules**			
AGEs	kidney damage; chronic inflammation; hypertension; atherosclerosis; dialysis amyloidosis	activity of NADPH oxidase; ROS release from mitochondria	[158,193]
AOPPs	kidney damage; chronic inflammation; hypertension; atherosclerosis; cardiomyopathy	activity of NADPH oxidase; ROS release from mitochondria	[153,154]
CMPF—a metabolite of furan fatty acid and a marker of fish oil intake	metabolism disruption; hepatopathy	glutathione metabolism (hepatocytes)	[194]
O-hydroxy hippuric acid	kidney damage	ROS generation (renal tubules)	[198]
P-cresol sulfate	kidney damage; chronic inflammation	ROS generation (leucocytes)	[199]
Indoxyl sulfate; Indole 3-acetic acid; Indoxyl-β-D-glucuronide	kidney damage; hypertension; atherosclerosis; calciphylaxis; prothrombotic effect	activity of NADPH oxidase	[132,191,199]
Kynurenine; 3-hydroxy kynurenine; Kynurenic acid; Quinolinic acid	anemia; hypertension; atherosclerosis; calciphylaxis; prothrombotic effect	activity of NADPH oxidase	[191,199]
Phenylacetic acid	chronic inflammation; hypertension; atherosclerosis; renal osteodystrophy	ROS generation (VSMC)	[194]
**Larger Middle Molecules (Molecular Weight > 500 Da)**			
IL-1β, IL-6, IL-8, IL-18, TNF-α	chronic inflammation; atherosclerosis	respiratory burst (neutrophils)	[105,200]
Ghrelin	water and electrolyte disturbances endocrine disorders atherosclerosis	ROS release from mitochondria	[194]
PTH	secondary hyperparathyroidism	ROS release from mitochondria	[94]
Leptin	chronic inflammation anemia hypertension; atherosclerosis; suppression of the immune system	ROS release from mitochondria	[201,202]
FGF-23	chronic inflammation hypertension atherosclerosis calciphylaxis cardiomyopathy	ROS release from mitochondria	[203]
